# Supplementary material for: Adherence to lipid-lowering guidelines for secondary prevention and potential reduction in CVD events in Swedish primary care: a cross-sectional study
Source: BMJ Open. 2020 Oct 10;10(10):e036920. doi: 10.1136/bmjopen-2020-036920 (PMC7549446; doi:10.1136/bmjopen-2020-036920)
Supplement: Supplementary data [file bmjopen-2020-036920supp001.pdf]

Supplementary information – Adherence to lipid-lowering guidelines for secondary prevention and potential reduction in CVD events in Swedish primary care – a cross-sectional study. Ödesjö et al

Table I. Sensitivity analyses

	Percentage estimated events	Percentage estimated events with LDL-C < 1.8 mmol/L	Reduction in number of events
**Model presented in the manuscript**	24.8%	20.4%	17.7%
**Data with BMI/smoking[1]**	22.1%	18.3%	17.1%
**Data with LDL-C[2]**	22.2%	18.3%	17.6%
**With SBP in model[3]**	23.9%	19.7%	17.7%
**With statin in model[4]**	24.6%	20.2%	18.0%

[1] Calculations based on the patients in the risk and study cohorts with information on BMI and smoking. The same risk model was applied as in the presented material as well as a model including BMI and smoking. Both models resulted in similar percentages.

[2] Calculations based on the patients in the risk and study cohorts with information on LDL-C. The same risk model applied as in the presented material.

[3] Calculations based on the same risk and study cohorts but with SBP added to the model, patients without information on SBP were thereby not included.

[4] Calculations based on the same risk and study cohorts but with statin added to the model.

Table II. Characteristics of patients in the study cohort divided by statin treatment or not.

Variable	No statin treatment (n= 11960)	Statin treatment (n= 25160)
Age	74.5 (11.0)	72.3 (9.5)
Sex (female)	5,529 (46.2%)	8,056 (32.0%)
Smoking	1,300 (12.5%)	2,921 (12.7%)
SBP (mmHg)	134.3 (17.0)	132.1 (15.6)
DBP (mmHg)	76.1 (11.2)	74.9 (10.4)
Total cholesterol (mmol/L)	5.2 (1.3)	4.2 (1.0)
LDL-C (mmol/L)	3.3 (1.1)	2.4 (0.8)
Triglyceride (mmol/L)	1.6 (0.9)	1.5 (0.9)
Hypertension	9,858 (82.4%)	21,011 (83.5%)
Diabetes	3488 (29.2%)	9056 (36.0%)
CHD	11,960 (100.0%)	25,160 (100.0%)
AMI	4,001 (33.5%)	12,741 (50.6%)
AMI past year	285 (2.4%)	1,259 (5.0%)
Stroke	1,315 (11.0%)	3,137 (12.5%)
Stroke past year	323 (2.7%)	969 (3.9%)
CVD	4,798 (40.1%)	14,385 (57.2%)

Heart failure	3,070 (25.7%)	5,818 (23.1%)
Atrial fibrillation	2,853 (23.9%)	5,404 (21.5%)
Dementia	589 (4.9%)	774 (3.1%)
ASA	5402 (45.2%)	18029 (71.7%)
Statin		25160 (100.0%)
Simvastatin		12820 (51.0%)
Pravastatin		286 (1.1%)
Atorvastatin		11424 (45.4%)
Rosuvastatin		998 (4.0%)
Other lipid lowering drugs	442 (3.7%)	789 (3.1%)
Ezetimib	277 (2.3%)	676 (2.7%)

Mean (SD) and frequencies (%). Abbreviations: SBP: Systolic blood pressure; DBP: Diastolic blood pressure; LDL-C: Low density lipoprotein cholesterol; CHD: coronary heart disease; AMI: acute myocardial infarction; CVD: cardiovascular disease; ASA: acetylic salicylic acid.
