# Supplementary material for: Adherence to lipid-lowering guidelines for secondary prevention and potential reduction in CVD events in Swedish primary care: a cross-sectional study
Source: BMJ Open. 2020 Oct 10;10(10):e036920. doi: 10.1136/bmjopen-2020-036920 (PMC7549446; doi:10.1136/bmjopen-2020-036920)
Supplement: Author's manuscript [file bmjopen-2020-036920.draft_revisions.pdf]

BMJ Open is committed to open peer review. As part of this commitment we make the peer review history of every article we publish publicly available.

When an article is published we post the peer reviewers' comments and the authors' responses online. We also post the versions of the paper that were used during peer review. These are the versions that the peer review comments apply to.

The versions of the paper that follow are the versions that were submitted during the peer review process. They are not the versions of record or the final published versions. They should not be cited or distributed as the published version of this manuscript.

BMJ Open is an open access journal and the full, final, typeset and author-corrected version of record of the manuscript is available on our site with no access controls, subscription charges or pay-per-view fees (http://bmjopen.bmj.com).

If you have any questions on BMJ Open's open peer review process please email info.bmjopen@bmj.com

BMJ Open

Adherence to lipid-lowering guidelines for secondary prevention and potential reduction in CVD events – an observational study in primary care

Journal:	*BMJ Open*
Manuscript ID	bmjopen-2020-036920
Article Type:	Original research
Date Submitted by the Author:	17-Jan-2020

Complete List of Authors:	Ödesjö, Helena; University of Gothenburg Department of Public Health and Community Medicine,<br>Björck, Staffan; Centre of Registers Västra Götaland<br>Franzén, Stefan; Centre of Registers Västra Götaland<br>Hjerpe, Per; University of Gothenburg Department of Public Health and Community Medicine<br>Manhem, Karin; University of Gothenburg Department of Molecular and Clinical Medicine<br>Rosengren, Annika; University of Gothenburg Department of Molecular and Clinical Medicine<br>Thorn, Jörgen; University of Gothenburg Department of Public Health and Community Medicine, Primary Health Care<br>Adamsson Eryd, Samuel; University of Gothenburg Department of Molecular and Clinical Medicine

Keywords:	Coronary heart disease < CARDIOLOGY, PRIMARY CARE, Cardiac Epidemiology < CARDIOLOGY, Lipid disorders < DIABETES & ENDOCRINOLOGY

SCHOLARONE™
Manuscripts

I, the Submitting Author has the right to grant and does grant on behalf of all authors of the Work (as defined in the below author licence), an exclusive licence and/or a non-exclusive licence for contributions from authors who are: i) UK Crown employees; ii) where BMJ has agreed a CC-BY licence shall apply, and/or iii) in accordance with the terms applicable for US Federal Government officers or employees acting as part of their official duties; on a worldwide, perpetual, irrevocable, royalty-free basis to BMJ Publishing Group Ltd ("BMJ") its licensees and where the relevant Journal is co-owned by BMJ to the co-owners of the Journal, to publish the Work in this journal and any other BMJ products and to exploit all rights, as set out in our licence.

The Submitting Author accepts and understands that any supply made under these terms is made by BMJ to the Submitting Author unless you are acting as an employee on behalf of your employer or a postgraduate student of an affiliated institution which is paying any applicable article publishing charge ("APC") for Open Access articles. Where the Submitting Author wishes to make the Work available on an Open Access basis (and intends to pay the relevant APC), the terms of reuse of such Open Access shall be governed by a Creative Commons licence – details of these licences and which Creative Commons licence will apply to this Work are set out in our licence referred to above.

Other than as permitted in any relevant BMJ Author's Self Archiving Policies, I confirm this Work has not been accepted for publication elsewhere, is not being considered for publication elsewhere and does not duplicate material already published. I confirm all authors consent to publication of this Work and authorise the granting of this licence.

For peer review only

Adherence to lipid-lowering guidelines for secondary prevention and potential reduction in CVD events – an observational study in primary care

H. Ödesjö[ab], S. Björck[d], S. Franzén[d], P. Hjerpe[ae], K. Manhem[c], A. Rosengren[c], J. Thorn[a], S. Adamsson Eryd[c]

[a]Department of Public Health and Community Medicine, Institute of Medicine, Sahlgrenska Academy, University of Gothenburg, Medicinaregatan 16, SE 405 30 Gothenburg, Sweden

[b]Primary Health Care, Region Västra Götaland, Närhälsan Tjörn Vårdcentral, Syster Ebbas väg, SE-471 94 Kållekärr, Sweden

[c]Department of Molecular and Clinical Medicine, Institute of Medicine, Sahlgrenska Academy, University of Gothenburg and Sahlgrenska University Hospital, Östra Hospital, Diagnosvägen 11, SE 416 50 Gothenburg, Sweden

[d]Centre of Registers Västra Götaland, Medicinaregatan 18G, SE 413 45 Gothenburg, Sweden

[e]R&D Centre Skaraborg Primary Care, Skövde, Långgatan 18, SE 541 30 Skövde, Sweden

Contact information:

Helena Ödesjö, MD, PhD student: helena.odesjo@vgregion.se, +46 73 561 78 23

(corresponding author, electronic reprints on demand)

(Staffan Björck, Stefan Franzén, Per Hjerpe, Karin Manhem, Annika Rosengren, Jörgen Thorn, Samuel Adamsson Eryd)

Presented as a poster on ESC Paris 2019. Part of a doctoral thesis 11/10 2019, http://hdl.handle.net/2077/60284

For peer review only

For peer review only - http://bmjopen.bmj.com/site/about/guidelines.xhtml

This work was supported by Närhälsan R&D Health Care, R&D Centre Gothenburg and Södra Bohuslän for postgraduate studies. This work was also supported by grants from the Swedish government under the Agreement concerning Research and Education of Doctors [grant number ALFGBG-717211], the Swedish Heart and Lung Foundation [grant number 2018-0366] and the Swedish Research Council [grant number 2018-02527].

Wordcount manuscript: 2 488

For peer review only

Abstract

Objectives

The protective effect of lipid lowering treatment for secondary prevention after coronary heart disease (CHD) has been well documented. Current guidelines recommend a target level for low density lipoprotein cholesterol (LDL-C) of ≤1.8 mmol/L. The aim was to describe adherence to guidelines and predict potential reductions in cardiovascular disease (CVD) events if patients: 1) with low-dose/less potent or no statin were treated with atorvastatin 80 mg, or 2) reached LDL-C ≤1.8 mmol/L.

Design

Cross-sectional.

Setting

Primary care in a large Swedish region.

Participants

37, 120 patients with diagnosis CHD in a Swedish regional primary care quality register (QregPV), by 31 December 2015.

Primary and secondary outcome measures

For peer review only

Proportion of patients on statin treatment and proportion of patients achieving LDL-C ≤1.8 mmol/L. Predicted number of CVD events calculated for a) current treatment, b) improved treatment and c) lowered LDL-C. Risk estimation modelling was based on 52,042 patients in the same register on January 2011 who had been followed for five years.

Results

Of 37,120 patients, 18% reached LDL-C ≤1.8 mmol/L and 32% were not on statin treatment. Based on individual risks, the estimated number of CVD events in the study group over 5 years was 9,209/37,120. If all patients without a statin or with less potent statin treatment were given atorvastatin 80 mg, a reduction of CVD events by 14% (7,901 vs 9,209) was predicted. If all patients achieved LDL-C ≤1.8 mmol/L the predicted number of events was reduced by 18% (7,577 vs 9,209).

Conclusion

One third of patients with CHD in primary care were not on lipid lowering treatment. By improving adherence to treatment guidelines, a substantial reduction in CVD events among patients with established CHD in primary care could be achieved.

For peer review only

Wordcount abstract: 283

Keywords

cardiovascular disease, guideline adherence, primary health care, secondary prevention, statins, Sweden

Strengths and limitations

We assessed adherence to lipid-lowering treatment guidelines in a regional primary care population of 57 341 patients with coronary heart disease (whereof 37 120 with information on LDL-C).

Information regarding other risk factors, primarily BMI and smoking, were missing to a large extent in the register.

Using data from the same register for patients followed for five years, a risk estimation model was created and applied to the study population in order to estimate the potential reduction in CVD events if guidelines were to be followed more extensively.

Ethical approval

The regional Ethical Review Board at the University of Gothenburg

For peer review only

For peer review only - http://bmjopen.bmj.com/site/about/guidelines.xhtml

Introduction

Lowering of low density lipoprotein cholesterol (LDL-C) by statin treatment in patients with coronary heart disease (CHD) effectively reduces the risk of recurrent cardiovascular disease (CVD) events[1, 2]. Current evidence indicates an almost linear relationship between LDL-C level and risk of CVD[3, 4]. Although CVD mortality has decreased by over 60 % over the past few decades in Sweden, it still accounts for one out of every three deaths[5].

Current European and Swedish guidelines recommend a treatment goal for LDL-C below 1.8 mmol/L in patients with established CHD[6, 7]. There has been a gradual improvement in the proportion of patients in Sweden reaching the LDL-C target one year after an acute myocardial infarction (AMI), and recent data suggests a figure of 65% in 2017[8]. Nevertheless, less is known about long-term adherence to current guidelines among patients with CHD in primary care or how an improvement in that regard affects morbidity.

The aim of this study was to describe adherence to guidelines and estimate the potential reduction in CVD events if all primary care patients with CHD were to improve in that regard.

For peer review only

Methods

We performed an observational cross-sectional register study of all primary care patients with CHD in a large Swedish region as of 31 December 2015.

Study basis

Västra Götaland (VGR) in south-west Sweden is a mixed urban and rural region with 1.7 million inhabitants (17% of the population). Since 2009, all individuals in the region have

For peer review only - http://bmjopen.bmj.com/site/about/guidelines.xhtml

been enrolled at a primary care centre. The region has about 200 centres that are publicly funded, whether publicly or privately operated. Primary care centres report individual data for all patients with CHD to a regional primary care quality register (QregPV).

Databases

This study proceeded from information obtained by linking data from QregPV, a regional administrative health care database (Vega), the National Patient Register (NPR), the Prescribed Drug Register and the Cause of Death Register.

QregPV contains information about all primary care patients, irrespective of age or severity of disease, with a diagnosis of hypertension and CHD in VGR, including blood pressure, lipid levels, smoking, height and weight recorded during appointments and reported on a monthly basis. Starting in 2009, reporting to the register has been mandatory for all primary care units in VGR.

Vega covers all primary care in VGR since 2000. Data include diagnoses, dates and professional categories. NPR covers all hospital discharge diagnoses in Sweden since 1987, as well as outpatient appointments in specialised care since 2001. This study included NPR data since 1997, when the International Classification of Disease 10th revision (ICD-10) was adopted. The Prescribed Drug Register contains information about all prescriptions filled since 1 July 2005. The Cause of Death Register consists of information about underlying and contributing causes of death since 1961.

All patients in QregPV with a diagnosis of CHD entered from 1 January 2010 to 31 December 2015 were included. Diagnoses were defined according to ICD-10: CHD I20-I25, AMI I21, stroke I61, I63 - I64, type 2 diabetes mellitus E10-14, heart failure I50 and atrial fibrillation and flutter I48. CVD was defined as acute myocardial infarction (AMI) or stroke.

For peer review only - http://bmjopen.bmj.com/site/about/guidelines.xhtml

For peer review only

Patients

Proceeding from the patient file, we created two cohorts:

- Study cohort (2015) – All patients with CHD in QregPV on 31 December 2015 (index date). Previous comorbidity until 31 December 2015 and prescriptions filled up to 120 days before the index date were collected from NPR, Vega and the Prescribed Drug Register.
- Risk estimation cohort (2011) - 52042 patients with CHD in QregPV on 1 January 2011 (index date). Previous comorbidity until the index date and new events corresponding to diagnoses of AMI and stroke until 31 December 2015 were added from NPR and Vega. Information about deaths were retrieved from the Cause of Death Register.

For both cohorts, medical data were collected in accordance with the last observation carried forward method. The latest entry of each variable was used up to 900 days before the index date.

Risk estimation

The risk estimation cohort was used to create a model for the risk of recurrent CVD and all-cause mortality based on individual data for age, sex, diabetes mellitus, history of heart failure and/or atrial fibrillation, stroke or AMI during the previous year and treatment with acetylic salicylic acid. Statin treatment was not included since it is one of the factors we assess. Smoking and BMI were not included due to a large number of missing values. Blood pressure was not included due to missing data in 2010 and the J-curved association described in observational studies[9].

For peer review only - http://bmjopen.bmj.com/site/about/guidelines.xhtml

Prediction

We calculated the individual risk of CVD and death from all causes, for patients in the study cohort, using the risk prediction model. We performed two separate adjusted predictions and evaluated the effect of:

- lowering LDL-C to 1.8 mmol/L for all patients with a higher level
- adding atorvastatin 40/80 mg to patients with no or less potent statin therapy and LDL-C > 1.8 mmol/L.

For risk reduction associated with lowering of LDL-C, data from Cholesterol Treatment Trialists' (CTT) collaboration were used. Overall risk reduction for any major vascular event was 22% (rate ratio 0.78; 95% CI 0.76-0.80) per mmol/L reduction in LDL-C based on almost 170,000 individuals[3]. Risk reduction values for separate outcomes in the CTT study differed. Risk reduction per mmol/L LDL-C reduction for all-cause mortality was 0.91 based on results from another CTT collaboration study[10]. Risk reduction with intensified statin treatment proceeded from expected percentage LDL-C lowering related to type of statin and dosage based on studies concerning statin effect on LDL-C level[11, 12].

Statistical methods

Descriptive statistics are presented as arithmetic mean and standard deviation for continuous variables and with frequencies and percentages for categorical variables. A Cox regression model was applied to the risk estimation cohort. The variables included in the model were age, sex, diabetes mellitus, history of heart failure, history of atrial fibrillation, treatment with acetyl salicylic acid and stroke or AMI during the previous year. Age was modelled by means of a smoothing spline-function with four degrees of freedom since the effect of age was not assumed to be linear.

For peer review only - http://bmjopen.bmj.com/site/about/guidelines.xhtml

Time to event was right censored at death as end of follow up for total mortality and time to CVD. The time-at-risk for each patient was defined as the time before the first event over a period of five years.

The survival function and risk of having experienced an event over the past 5 years were calculated. Summing up individual risks yielded the total number of individuals predicted to have experienced an event over the past 5 years. Adjustments were made such that the cumulative hazard was recalculated based on an expected reduced risk for events related to lowering of LDL-C directly or expected lowering as a result of more intense statin treatment. The cumulative hazard of an event was reduced by 22% per mmol/L LDL-C lowering. This model did not take competing risks into account, assuming that all patients were alive after five years if they did not suffer an event.

The statistical analyses used R 3.4.0 and SAS version 9.4 (SAS Institute, Cary, NC).

Patient and public involvement

Patients or the public were not involved in the design, or conduct, or reporting, or dissemination plans of our research.

Results

Descriptive statistics for the study cohort are shown in

Table 1. There was a total of 57,341 patients, 37,120 of whom had information about LDL-C at baseline. Fewer than 20% (6,747/37,120) achieved the LDL-C target of ≤ 1.8 mmol/L. Among the non-controlled patients, with LDL-C > 1.8 mmol/L, an average LDL-C reduction of 1.2 mmol/L (40%) would be required to reach the LDL-C target. The subset of patients with AMI or stroke in the previous year had a mean age of 74.2 (10.5); 78% received statin treatment and 25% reached the LDL-C target.

For peer review only - http://bmjopen.bmj.com/site/about/guidelines.xhtml

Table 1. Characteristics of patients in the study cohort.

Variable	All patients (n = 37,120)	LDL-C <= 1.8 mmol/L (n= 6,747)	LDL-C > 1.8 mmol/L (n= 30,373)
Age	73.0 (10.1)	73.2 (9.9)	73.0 (10.1)
Sex (female)	13585 (36.6%)	1897 (28.1%)	11688 (38.5%)
Smoking	4221 (12.7%)	755 (12.3%)	3466 (12.7%)
SBP (mmHg)	132.8 (16.1)	130.6 (15.6)	133.3 (16.2)
DBP (mmHg)	75.3 (10.7)	73.9 (10.7)	75.6 (10.7)
Total cholesterol (mmol/L)	4.5 (1.2)	3.4 (0.7)	4.8 (1.1)
LDL-C (mmol/L)	2.7 (1.0)	1.5 (0.3)	3.0 (0.9)
Triglyceride (mmol/L)	1.6 (0.9)	1.5 (1.0)	1.6 (0.9)
Hypertension	30869 (83.2%)	5649 (83.7%)	25220 (83.0%)
Diabetes	12544 (33.8%)	3116 (46.2%)	9428 (31.0%)
CHD	37120 (100.0%)	6747 (100.0%)	30373 (100.0%)
AMI	16742 (45.1%)	3740 (55.4%)	13002 (42.8%)
AMI past year	1544 (4.2%)	441 (6.5%)	1103 (3.6%)
Stroke	4452 (12.0%)	921 (13.7%)	3531 (11.6%)
Stroke past year	1292 (3.5%)	266 (3.9%)	1026 (3.4%)
CVD	19183 (51.7%)	4177 (61.9%)	15006 (49.4%)
Heart failure	8888 (23.9%)	1951 (28.9%)	6937 (22.8%)
Atrial fibrillation	8257 (22.2%)	1807 (26.8%)	6450 (21.2%)
Dementia	1363 (3.7%)	224 (3.3%)	1139 (3.8%)
ASA	23431 (63.1%)	4426 (65.6%)	19005 (62.6%)
Statin	25160 (67.8%)	5735 (85.0%)	19425 (64.0%)

For peer review only - http://bmjopen.bmj.com/site/about/guidelines.xhtml

Variable	All patients (n = 37,120)	LDL-C <= 1.8 mmol/L (n= 6,747)	LDL-C > 1.8 mmol/L (n= 30,373)
Simvastatin	12820 (34.5%)	2810 (41.6%)	10010 (33.0%)
Pravastatin	286 (0.8%)	20 (0.3%)	266 (0.9%)
Atorvastatin	11424 (30.8%)	2723 (40.4%)	8701 (28.6%)
Rosuvastatin	998 (2.7%)	235 (3.5%)	763 (2.5%)
Other lipid lowering drugs	1231 (3.3%)	227 (3.4%)	1004 (3.3%)
Ezetimib	953 (2.6%)	175 (2.6%)	778 (2.6%)

Abbreviations: SBP: Systolic blood pressure; DBP: Diastolic blood pressure; LDL-C: Low density lipoprotein cholesterol; CHD: coronary heart disease; AMI: acute myocardial infarction; CVD: cardiovascular disease; ASA: acetylic salicylic acid.

Statin treatment was most prevalent for age 50-75, approximately 70% of whom filled a prescription, see Figure 1. Prescriptions filled for statins diminish rapidly after the age of 80.

Figure 1.

LDL-C for uncontrolled patients was predicted to decrease from a mean of 3.0 to 2.2 mmol/L if all patients received atorvastatin 40 mg and to 2.0 mmol/L with atorvastatin 80 mg. For all patients in the study cohort the corresponding levels were 2.7 to 2.1 and 1.9 mmol/L, see LDL-C and predicted LDL-C distribution in Figure 2.

Figure 2.

For peer review only - http://bmjopen.bmj.com/site/about/guidelines.xhtml

Total predicted CVD events for 5 years in the study cohort was 9,209 (24.8%), see Figure 3. A decrease of LDL-C to the target (≤ 1.8 mmol/l) or intensified statin treatment (all patients with less efficient therapy receive atorvastatin 80 mg) would result in a reduction of expected CVD events for 5 years by 1,632 and 1,308. This would result in a decreased event risk of 18% and 14%. Including only the patients with LDL-C > 1.8, the corresponding reductions were 22% and 18%. All-cause mortality for 5 years declined by 6.4 % (534/8,344) when LDL-C was lowered to ≤ 1.8 mmol/L.

Figure 3.

Sensitivity analyses by adding variables showed that the model was robust. When BMI and smoking status were included, the number of patients in the risk estimation cohort was reduced, from 52,042 to 9,254. The proportion of patients who were predicted to suffer a CVD event was decreased from 25% to 22% but the relative reduction in number of events was the same. The results were similar without including BMI and smoking status in the model. The same was true if the risk estimation model was based on the 16,577 patients with LDL-C (although LDL-C was not in the model). When systolic blood pressure was included in the model, the risk estimation cohort was decreased to 35,989 and the outcomes were relatively unchanged. Inclusion of statin treatment (0/1) in the model did not lead to any changes in the results.

The proportion of patients predicted to experience a CVD event was 24.6 % (2,937/11,960) among non-statin users and 24.9 % (6,271/25,160) among statin users, see Figure 4. If all patients were prescribed 80 mg atorvastatin, the predicted proportion to experience a CVD event was reduced by 32.9 % (967) among non-statin users and 5.4 % (339) among statin users.

Figure 4.

For peer review only - http://bmjopen.bmj.com/site/about/guidelines.xhtml

For peer review only

Other lipid-lowering drugs were rarely used, 953/37,120 (2.6%) only 442 of whom were not also on statin. Patients receiving other lipid-lowering drugs had slightly higher LDL-C than the rest of the study population.

Discussion

Summary

This study shows that the number of CVD events could be substantially reduced if patients with no/low-dose statin were given atorvastatin 80 mg and even more so if all patients reached the LDL-C target. 1/3 of our study population had no statin treatment and fewer than 1/5 reached the recommended LDL-C target of 1.8 mmol/L. The largest gain would result from initiating treatment in patients who are not receiving statin.

Strengths and limitations

The main strength of our study was the large number of primary care patients with CHD. Previous studies were often based on a post-MI population from secondary care registers. Another strength is the completeness of data concerning comorbidities and filled prescriptions. We based our risk model calculations on the same regional population as the predictions.

Information about LDL-C level was missing for many patients in the study cohort. These patients were older, diagnosed more often with atrial fibrillation and heart failure, less with diabetes and not as likely to be receiving statin treatment. Inclusion of them would probably have increased the predicted number of saved events. BMI, smoking and blood pressure were not included in the model due to the quantity of missing values in the risk estimation cohort. Sensitivity analyses showed that the results remained approximately

For peer review only - http://bmjopen.bmj.com/site/about/guidelines.xhtml

For peer review only

the same without these variables. We assumed that all patients benefit proportionally from better adherence and improved treatment.

We did not take competing risks into account and assumed that no patient had died from other causes during the follow-up period. Patients in our risk estimation cohort who died before a potential event were right censored and 9.2% died from other causes without a previous event. Our model appears to slightly overestimate the number of CVD events but the percentage decrease is not necessarily affected.

Comparison with existing literature

A recent Swedish study described short-term adherence to statins and potential effects of improved treatment among 5,904 post-MI patients[13]. The study found an estimated 805-2,262 CVD events in over 10 years among those who did not reach LDL-C target and predicted that a reduction of LDL-C to 1.8 mmol/L would lower the number by 132-343 (15-16%). The patients in that study were younger, which might explain the smaller predicted reduction in events.

LDL-C is monitored one year after AMI in the Swedish national quality register (SEPHIA). In 2015, mean LDL-C was 1.99 mmol/L and 51% had LDL-C < 1.8 mmol/L[14]. Short-term adherence to lipid lowering treatment after AMI is gradually improving in Sweden[8]. A Norwegian study of 42,707 patients discharged from hospital after AMI in 2009-2013 reported that 84% were being treated with statins one year later[15]. Mean LDL-C was 2.7 mmol/L in our study cohort and only 18% had LDL-C ≤ 1.8 mmol/L also including those younger than 75 as those in SEPHIA. This difference between improved treatment in the short term and our finding of poor control in the long term may be due to poorer adherence to lipid-lowering treatment over time and the fact that new guidelines are applied to patients with a recent AMI event. Over the past few

For peer review only - http://bmjopen.bmj.com/site/about/guidelines.xhtml

years, statin treatment has proven to be safe and effective among the aged as well, but the proportion of patients in our study receiving it diminishes after the age of 75[2].

Our cohort consists of primary care patients with a CHD diagnosis. It has been shown that patients in primary care with known CVD from diagnoses in hospital but without a registered one in primary care receive less statin treatment[16]. In light of these results, our study presumably underestimates the potential event reduction by means of improved treatment.

The reasons for non-adherence to guidelines may depend on the physician, patient or organisational factors. A recent Swedish study showed that patients with concomitant use of other cardio-protective medications or smoking had higher adherence to refill and lipid-lowering treatment[17]. Two Danish studies found adherence to statin treatment to be lower among patients with a more risk-seeking attitude and physicians who did not assess treatment after initiation[18, 19]. The reasons that physicians do not adhere to lipid-lowering guidelines may be the same as for the blood pressure target: acceptance of higher values than recommended, competing medical problems, etc.[20].

Implications for research and/or practice

The treatment gap for lipid-lowering therapy in a primary care population with established CHD was large, representing a significant potential to reduce the number of events if treatment guidelines were to be followed to a greater extent.

Funding

The authors disclosed receipt of the following financial support for the research, authorship, and/or publication of this article: This work was supported by Närhälsan R&D Health Care, R&D Centre Gothenburg and Södra Bohuslän for postgraduate studies. This

For peer review only - http://bmjopen.bmj.com/site/about/guidelines.xhtml

work was also supported by grants from the Swedish government under the Agreement concerning Research and Education of Doctors [grant number ALFGBG-717211], the Swedish Heart and Lung Foundation [grant number 2018-0366] and the Swedish Research Council [grant number 2018-02527].

Declaration of Conflicting Interests

SAE is employed by AstraZeneca.

Author contributions

HÖ, SB, SF, PH, KM, AR and SAE contributed to the conception and design of the work. All authors contributed to the acquisition, analysis, or interpretation of data for the work. HÖ drafted the manuscript. All authors critically revised the manuscript. All authors gave final approval and agree to be accountable for all aspects of work ensuring integrity and accuracy.

Data sharing statement

No additional unpublished data are available.

For peer review only

References

1. Lind L, Sundstrom J, Arnlov J, et al. Impact of Aging on the Strength of Cardiovascular Risk Factors: A Longitudinal Study Over 40 Years. *J Am Heart Assoc.* 2018;7(1).

2. Cholesterol Treatment Trialists C, Armitage J, Baigent C, et al. Efficacy and safety of statin therapy in older people: a meta-analysis of individual participant data from 28 randomised controlled trials. *Lancet*. 2019;393(10170):407-15.

3. Cholesterol Treatment Trialists C, Baigent C, Blackwell L, et al. Efficacy and safety of more intensive lowering of LDL cholesterol: a meta-analysis of data from 170,000 participants in 26 randomised trials. *Lancet*. 2010;376(9753):1670-81.

4. Ference BA, Ginsberg HN, Graham I, et al. Low-density lipoproteins cause atherosclerotic cardiovascular disease. 1. Evidence from genetic, epidemiologic, and clinical studies. A consensus statement from the European Atherosclerosis Society Consensus Panel. *Eur Heart J*. 2017;38(32):2459-72.

5. Statistik om dödsorsaker 2017 [Cause of Death statistics 2017]. https://www.socialstyrelsen.se/Lists/Artikelkatalog/Attachments/21101/2018-10-17.pdf: Social Board of Health and Welfare; 2018.

6. To prevent atherosclerotic cardiovascular disease with drugs - treatment recommendations. Stockholm. Sweden: Medical Product Agency; [updated 2014; cited 2018 7/12]. Available from: https://lakemedelsverket.se/upload/halso-och-sjukvard/behandlingsrekommendationer/Att_forebygga_aterosklerotisk_hjart-karlsjukdom_med%20_lakemedel_behandlingsrekommendation.pdf.

7. Authors/Task Force M, Piepoli MF, Hoes AW, et al. 2016 European Guidelines on cardiovascular disease prevention in clinical practice: The Sixth Joint Task Force of the European Society of Cardiology and Other Societies on Cardiovascular Disease Prevention in

For peer review only - http://bmjopen.bmj.com/site/about/guidelines.xhtml

Clinical Practice (constituted by representatives of 10 societies and by invited experts): Developed with the special contribution of the European Association for Cardiovascular Prevention & Rehabilitation (EACPR). *Eur J Prev Cardiol*. 2016;23(11):NP1-NP96.

8. Swedeheart. SWEDEHEART Annual Report 2017. Stockholm; 2017.

9. Adamsson Eryd S, Gudbjornsdottir S, Manhem K, et al. Blood pressure and complications in individuals with type 2 diabetes and no previous cardiovascular disease: national population based cohort study. *Bmj*. 2016;354:i4070.

10. Cholesterol Treatment Trialists C, Fulcher J, O'Connell R, et al. Efficacy and safety of LDL-lowering therapy among men and women: meta-analysis of individual data from 174,000 participants in 27 randomised trials. *Lancet*. 2015;385(9976):1397-405.

11. Law MR, Wald NJ, Rudnicka AR. Quantifying effect of statins on low density lipoprotein cholesterol, ischaemic heart disease, and stroke: systematic review and meta-analysis. *BMJ*. 2003;326(7404):1423.

12. NICE. NICE Guidance - Cardiovascular disease: risk assessment and reduction, including lipid modification London: The National Institute for Health and Care Excellence (NICE); 2014 [updated 2016; cited 2018 14/11]. Available from: https://www.nice.org.uk/guidance/cg181/chapter/appendix-a-grouping-of-statins.

13. Journath G, Hambraeus K, Hagstrom E, et al. Predicted impact of lipid lowering therapy on cardiovascular and economic outcomes of Swedish atherosclerotic cardiovascular disease guideline. *BMC Cardiovasc Disord*. 2017;17(1):224.

14. SWEDEHEART. SWEDEHEART Annual report 2015. Stockholm; 2015.

15. Halvorsen S, Jortveit J, Hasvold P, et al. Initiation of and long-term adherence to secondary preventive drugs after acute myocardial infarction. *BMC Cardiovasc Disord*. 2016;16:115.

For peer review only - http://bmjopen.bmj.com/site/about/guidelines.xhtml

16. Dahlgren C, Geary L, Hasselstrom J, et al. Recording a diagnosis of stroke, transient ischaemic attack or myocardial infarction in primary healthcare and the association with dispensation of secondary preventive medication: a registry-based prospective cohort study. *BMJ open*. 2017;7(9):e015723.

17. Karlsson SA, Hero C, Eliasson B, et al. Refill adherence and persistence to lipid-lowering medicines in patients with type 2 diabetes: A nation-wide register-based study. *Pharmacoepidemiol Drug Saf*. 2017;26(10):1220-32.

18. Barfoed BL, Paulsen MS, Christensen PM, et al. Associations between patients' adherence and GPs' attitudes towards risk, statin therapy and management of non-adherence--a survey and register-based study. *Family practice*. 2016;33(2):140-7.

19. Barfoed BL, Paulsen MS, Christensen PM, et al. Associations between patients' risk attitude and their adherence to statin treatment - a population based questionnaire and register study. *BMC family practice*. 2016;17:28.

20. Oliveria SA, Lapuerta P, McCarthy BD, et al. Physician-related barriers to the effective management of uncontrolled hypertension. *Arch Intern Med*. 2002;162(4):413-20.

For peer review only

Legends

Figure 1. Statin treatment by age. Included ages with more than 100 observations. The number of patients below the age of 50 are very few (603/37120) as are the patients over the age of 90 (925/37120).

Figure 2. LDL-C distribution in all patients in the study cohort with current treatment and adjusted treatment defined as if all patients received atorvastatin 80 mg (if less intense treatment before and LDL-C > 1.8 mmol/L).

Figure 3. Predicted number of events in the study cohort (37 120 patients) with registered levels of LDL-C and current treatment as well as when modelling a reduction in LDL-C to 1.8 mmol/L for all patients with higher LDL-C or an intensified statin lowering treatment with 80 mg atorvastatin for those with a less efficient treatment.

Figure 4. Predicted number of events in the study cohort with registered levels of LDL-C and current treatment separated in patients with and without current statin treatment and the predicted number of events when applying a more intense statin treatment to all patients with a less efficient treatment.

For peer review only - http://bmjopen.bmj.com/site/about/guidelines.xhtml

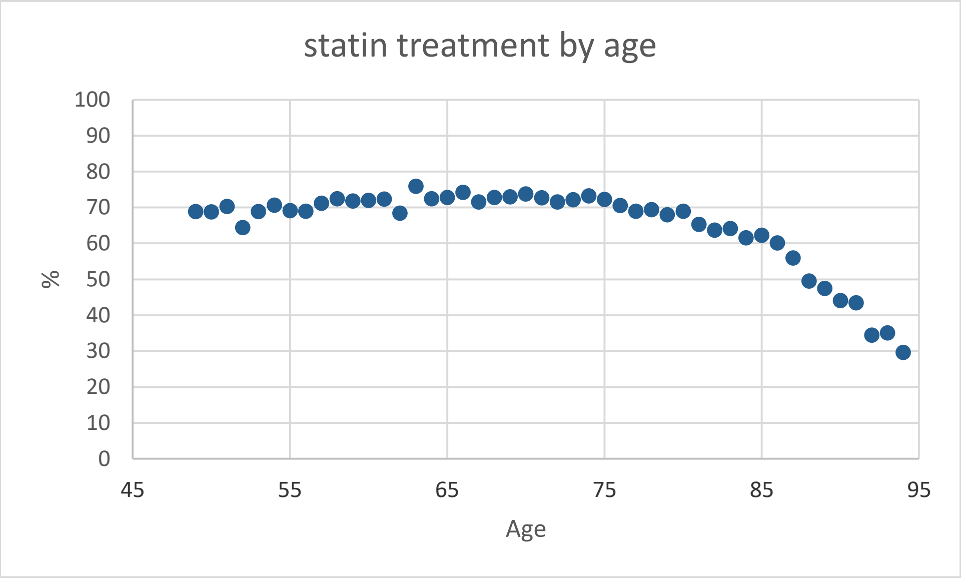

peer review only

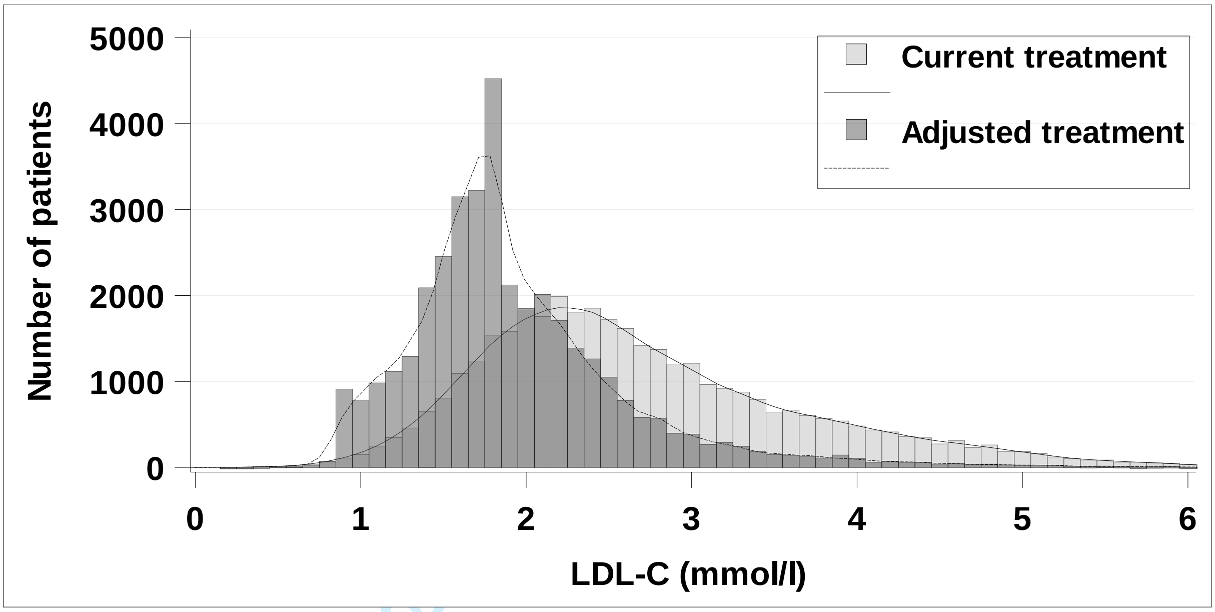

For peer review only

For peer review only - http://bmjopen.bmj.com/site/about/guidelines.xhtml

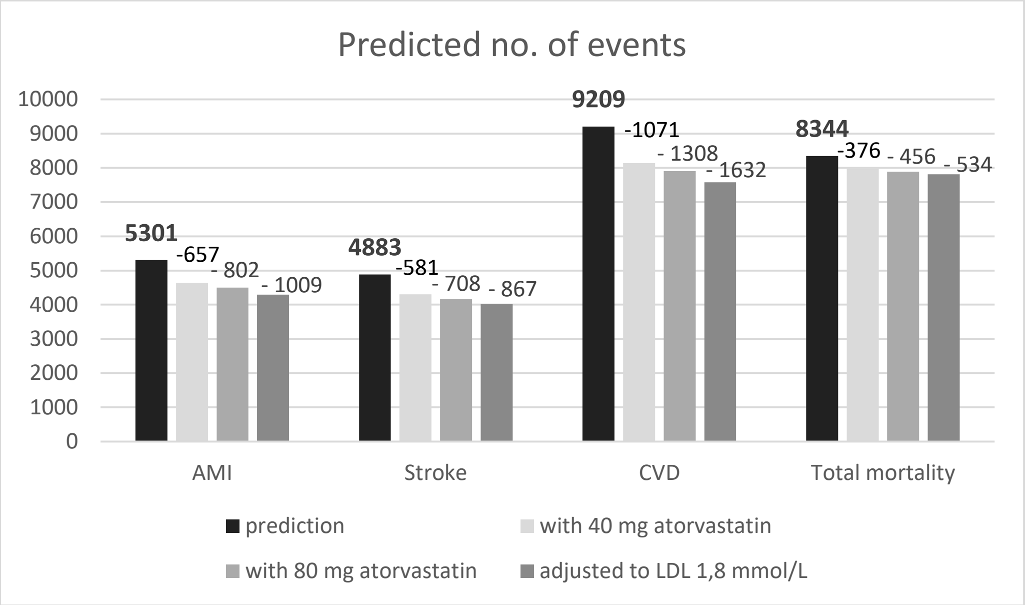

For peer review only - http://bmjopen.bmj.com/site/about/guidelines.xhtml

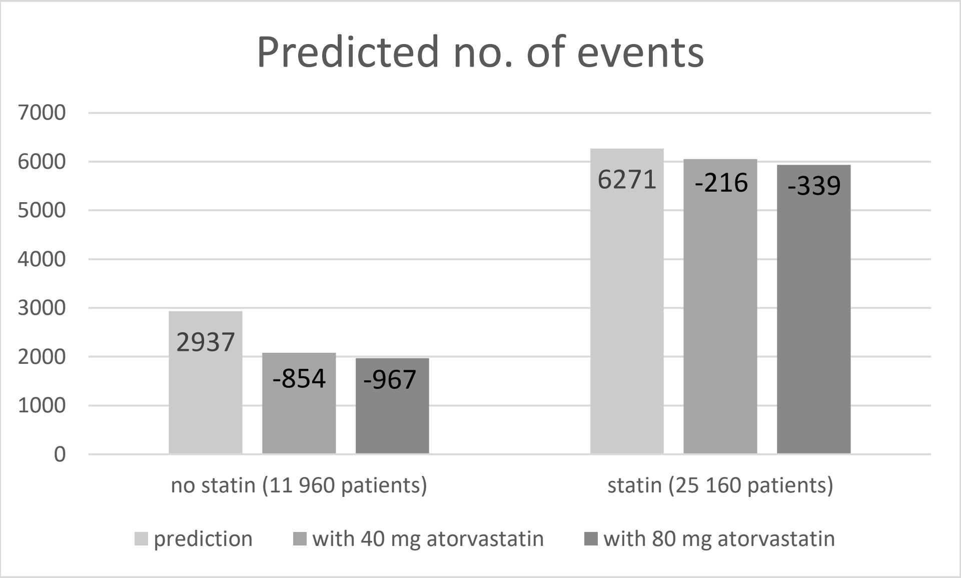
Predicted no. of events
7000
6000
5000
4000
3000
2000
1000
0
2937
-854
-967
6271
-216
-339
no statin (11 960 patients)
statin (25 160 patients)
prediction
with 40 mg atorvastatin
with 80 mg atorvastatin

1 (1)

STROBE 2007 (v4) Statement—Checklist of items that should be included in reports of *cross-sectional studies*

Section/Topic	Item #	Recommendation	Reported on page #
**Title and abstract**	1	(*a*) Indicate the study's design with a commonly used term in the title or the abstract	Yes
		(*b*) Provide in the abstract an informative and balanced summary of what was done and what was found	Yes
**Introduction**			
Background/rationale	2	Explain the scientific background and rationale for the investigation being reported	Yes
Objectives	3	State specific objectives, including any prespecified hypotheses	Yes
**Methods**			
Study design	4	Present key elements of study design early in the paper	Yes
Setting	5	Describe the setting, locations, and relevant dates, including periods of recruitment, exposure, follow-up, and data collection	Yes
Participants	6	(*a*) Give the eligibility criteria, and the sources and methods of selection of participants	Yes
Variables	7	Clearly define all outcomes, exposures, predictors, potential confounders, and effect modifiers. Give diagnostic criteria, if applicable	Yes
Data sources/ measurement	8*	For each variable of interest, give sources of data and details of methods of assessment (measurement). Describe comparability of assessment methods if there is more than one group	Yes
Bias	9	Describe any efforts to address potential sources of bias	*Yes*
Study size	10	Explain how the study size was arrived at	
Quantitative variables	11	Explain how quantitative variables were handled in the analyses. If applicable, describe which groupings were chosen and why	Yes
Statistical methods	12	(*a*) Describe all statistical methods, including those used to control for confounding	Yes
		(*b*) Describe any methods used to examine subgroups and interactions	NA
		(*c*) Explain how missing data were addressed	Yes
		(*d*) If applicable, describe analytical methods taking account of sampling strategy	NA
		(*e*) Describe any sensitivity analyses	Yes
**Results**			

For peer review only

Participants	13*	(a) Report numbers of individuals at each stage of study—eg numbers potentially eligible, examined for eligibility, confirmed eligible, included in the study, completing follow-up, and analysed	Yes
		(b) Give reasons for non-participation at each stage	Yes
		(c) Consider use of a flow diagram	
Descriptive data	14*	(a) Give characteristics of study participants (eg demographic, clinical, social) and information on exposures and potential confounders	Yes
		(b) Indicate number of participants with missing data for each variable of interest	Yes
Outcome data	15*	Report numbers of outcome events or summary measures	Yes
Main results	16	(*a*) Give unadjusted estimates and, if applicable, confounder-adjusted estimates and their precision (eg, 95% confidence interval). Make clear which confounders were adjusted for and why they were included	NA
		(*b*) Report category boundaries when continuous variables were categorized	NA
		(*c*) If relevant, consider translating estimates of relative risk into absolute risk for a meaningful time period	
Other analyses	17	Report other analyses done—eg analyses of subgroups and interactions, and sensitivity analyses	Yes
**Discussion**			
Key results	18	Summarise key results with reference to study objectives	Yes
Limitations	19	Discuss limitations of the study, taking into account sources of potential bias or imprecision. Discuss both direction and magnitude of any potential bias	Yes
Interpretation	20	Give a cautious overall interpretation of results considering objectives, limitations, multiplicity of analyses, results from similar studies, and other relevant evidence	Yes
Generalisability	21	Discuss the generalisability (external validity) of the study results	Yes
**Other information**			
Funding	22	Give the source of funding and the role of the funders for the present study and, if applicable, for the original study on which the present article is based	Yes

*Give information separately for cases and controls in case-control studies and, if applicable, for exposed and unexposed groups in cohort and cross-sectional studies.

Note: An Explanation and Elaboration article discusses each checklist item and gives methodological background and published examples of transparent reporting. The STROBE checklist is best used in conjunction with this article (freely available on the Web sites of PLoS Medicine at http://www.plosmedicine.org/, Annals of Internal Medicine at http://www.annals.org/, and Epidemiology at http://www.epidem.com/). Information on the STROBE Initiative is available at www.strobe-statement.org.

For peer review only

BMJ Open

Adherence to lipid-lowering guidelines for secondary prevention and potential reduction in CVD events in Swedish primary care – a cross-sectional study

Journal:	*BMJ Open*
Manuscript ID	bmjopen-2020-036920.R1
Article Type:	Original research
Date Submitted by the Author:	15-Jun-2020
Complete List of Authors:	Ödesjö, Helena; University of Gothenburg Sahlgrenska Academy, Primary Health Care, School of Public Health and Community Medicine, Institute of Medicine<br>Björck, Staffan; Centre of Registers Västra Götaland<br>Franzén, Stefan; Centre of Registers Västra Götaland<br>Hjerpe, Per; University of Gothenburg Sahlgrenska Academy, Primary Health Care, School of Public Health and Community Medicine, Institute of Medicine<br>Manhem, Karin; University of Gothenburg Department of Molecular and Clinical Medicine<br>Rosengren, Annika; University of Gothenburg Department of Molecular and Clinical Medicine<br>Thorn, Jörgen; University of Gothenburg Sahlgrenska Academy, Primary Health Care, School of Public Health and Community Medicine, Institute of Medicine<br>Adamsson Eryd, Samuel; University of Gothenburg Department of Molecular and Clinical Medicine
**Primary Subject Heading**:	General practice / Family practice
Secondary Subject Heading:	Cardiovascular medicine, General practice / Family practice
Keywords:	Coronary heart disease < CARDIOLOGY, PRIMARY CARE, Cardiac Epidemiology < CARDIOLOGY, Lipid disorders < DIABETES & ENDOCRINOLOGY

SCHOLARONE™
Manuscripts

I, the Submitting Author has the right to grant and does grant on behalf of all authors of the Work (as defined in the below author licence), an exclusive licence and/or a non-exclusive licence for contributions from authors who are: i) UK Crown employees; ii) where BMJ has agreed a CC-BY licence shall apply, and/or iii) in accordance with the terms applicable for US Federal Government officers or employees acting as part of their official duties; on a worldwide, perpetual, irrevocable, royalty-free basis to BMJ Publishing Group Ltd ("BMJ") its licensees and where the relevant Journal is co-owned by BMJ to the co-owners of the Journal, to publish the Work in this journal and any other BMJ products and to exploit all rights, as set out in our licence.

The Submitting Author accepts and understands that any supply made under these terms is made by BMJ to the Submitting Author unless you are acting as an employee on behalf of your employer or a postgraduate student of an affiliated institution which is paying any applicable article publishing charge ("APC") for Open Access articles. Where the Submitting Author wishes to make the Work available on an Open Access basis (and intends to pay the relevant APC), the terms of reuse of such Open Access shall be governed by a Creative Commons licence – details of these licences and which Creative Commons licence will apply to this Work are set out in our licence referred to above.

Other than as permitted in any relevant BMJ Author's Self Archiving Policies, I confirm this Work has not been accepted for publication elsewhere, is not being considered for publication elsewhere and does not duplicate material already published. I confirm all authors consent to publication of this Work and authorise the granting of this licence.

For peer review only

Adherence to lipid-lowering guidelines for secondary prevention and potential reduction in CVD events in Swedish primary care – a cross-sectional study

H. Ödesjö[ab], S. Björck[d], S. Franzén[d], P. Hjerpe[ae], K. Manhem[c], A. Rosengren[c], J. Thorn[a], S. Adamsson Eryd[c]

[a]Primary Health Care, School of Public Health and Community Medicine, Institute of Medicine, Sahlgrenska Academy, University of Gothenburg, SE 405 30 Gothenburg, Sweden

[b]Primary Health Care, Region Västra Götaland, Närhälsan Tjörn Vårdcentral, Syster Ebbas väg, SE-471 94 Kållekärr, Sweden

[c]Department of Molecular and Clinical Medicine, Institute of Medicine, Sahlgrenska Academy, University of Gothenburg and Sahlgrenska University Hospital, Östra Hospital, Diagnosvägen 11, SE 416 50 Gothenburg, Sweden

[d]Centre of Registers Västra Götaland, Medicinaregatan 18G, SE 413 45 Gothenburg, Sweden

[e]R&D Centre Skaraborg Primary Care, Skövde, Regionens hus, SE54130 Skövde, Sweden

Contact information:

Helena Ödesjö, MD, PhD student: helena.odesjo@vgregion.se, +46 73 561 78 23

(corresponding author, electronic reprints on demand)

(Staffan Björck, Stefan Franzén, Per Hjerpe, Karin Manhem, Annika Rosengren, Jörgen Thorn, Samuel Adamsson Eryd)

Presented as a poster on ESC Paris 2019. Part of a doctoral thesis 11/10 2019, http://hdl.handle.net/2077/60284

For peer review only

For peer review only - http://bmjopen.bmj.com/site/about/guidelines.xhtml

This work was supported by Närhälsan R&D Health Care, R&D Centre Gothenburg and Södra Bohuslän for postgraduate studies. This work was also supported by grants from the Swedish government under the Agreement concerning Research and Education of Doctors [grant number ALFGBG-717211], the Swedish Heart and Lung Foundation [grant number 2018-0366] and the Swedish Research Council [grant number 2018-02527].

Wordcount manuscript: 3,229

For peer review only - http://bmjopen.bmj.com/site/about/guidelines.xhtml

Abstract

Objectives

The protective effect of lipid lowering treatment for secondary prevention after coronary heart disease (CHD) has been well documented. Current guidelines recommend a target level for low density lipoprotein cholesterol (LDL-C) of ≤1.8 mmol/L. The aim was to describe adherence to guidelines and predict potential reductions in cardiovascular disease (CVD) events if patients: 1) with low-dose/less potent or no statin were treated with atorvastatin 80 mg, or 2) reached LDL-C ≤1.8 mmol/L.

Design

Cross-sectional.

Setting

Primary care in a large Swedish region.

Participants

37,120 patients with diagnosis CHD in a Swedish regional primary care quality register (QregPV), by 31 December 2015.

Primary and secondary outcome measures

For peer review only

For peer review only - http://bmjopen.bmj.com/site/about/guidelines.xhtml

Proportion of patients on statin treatment and proportion of patients achieving LDL-C ≤1.8 mmol/L. Predicted number of CVD events calculated for a) current treatment, b) improved treatment and c) lowered LDL-C, based upon applying rate reductions from a meta-analysis of RCTs to the potentially undertreated population. Risk estimation modelling was based on 52,042 patients in the same register on January 2011 who had been followed for five years.

Results

Of 37,120 patients, 18% reached LDL-C ≤1.8 mmol/L and 32% were not on statin treatment. Based on individual risks, the estimated number of CVD events in the study group over 5 years was 9,209/37,120. If all patients without a statin or with less potent statin treatment were given atorvastatin 80 mg, a reduction of CVD events by 14% (7,901 vs 9,209) was predicted. If all patients achieved LDL-C ≤1.8 mmol/L the predicted number of events was reduced by 18% (7,577 vs 9,209).

Conclusion

One third of patients with CHD in primary care were not on lipid lowering treatment. By improving adherence to treatment guidelines, a substantial reduction in CVD events among patients with established CHD in primary care could be achieved.

For peer review only

Wordcount abstract: 297

Keywords

cardiovascular disease, guideline adherence, primary health care, secondary prevention, statins, Sweden

Strengths and limitations of this study

The study was based on all patients with coronary heart disease in a Swedish region.

Data on comorbidities, filled prescriptions and outcomes were available for all patients.

Risk model calculations were based on the same regional population as the predictions.

There were missing data regarding BMI and smoking, known risk factors for cardiovascular disease.

We had no information on reasons for no statin treatment or low dose treatment.

Ethical approval

The regional Ethical Review Board at the University of Gothenburg

For peer review only

Introduction

Lowering of low density lipoprotein cholesterol (LDL-C) by statin treatment in patients with coronary heart disease (CHD) effectively reduces the risk of recurrent cardiovascular disease (CVD) events irrespective of age[1, 2]. Current evidence indicates an almost linear relationship between LDL-C level and risk of CVD[3, 4]. Although CVD mortality has decreased by over 60 % over the past few decades in Sweden, it still accounts for one out of every three deaths[5].

Low adherence to statin treatment is associated with a greater risk of death and new CVD events[6, 7]. The first years after a cardiovascular event, adherence to secondary preventive treatment is still high but diminishes over time [8, 9]. Adverse events are rare but nevertheless pose barriers for statin treatment both in patients and physicians[10-12].

Current European and Swedish guidelines recommend a treatment goal for LDL-C below 1.8 mmol/L in patients with established CHD[13, 14]. There has been a gradual improvement in the proportion of patients in Sweden reaching the LDL-C target one year after an acute myocardial infarction (AMI), and recent data suggests a figure of 65% in 2017[15]. Nevertheless, less is known about long-term adherence to current guidelines among patients with CHD in primary care or how an improvement in that regard affects morbidity.

The aim of this study was to describe adherence to guidelines and estimate the potential reduction in CVD events if all primary care patients with CHD were to improve in that regard.

For peer review only

For peer review only - http://bmjopen.bmj.com/site/about/guidelines.xhtml

Methods

We performed an observational cross-sectional register study of all primary care patients with CHD in a large Swedish region as of 31 December 2015.

Study basis

Västra Götaland (VGR) in south-west Sweden is a mixed urban and rural region with 1.7 million inhabitants (17% of the population). Since 2009, all individuals in the region have been enrolled at a primary care centre. The region has about 200 centres that are publicly funded, whether publicly or privately operated. Primary care centres report individual data for all patients with CHD to a regional primary care quality register (QregPV).

For peer review only

Databases

This study proceeded from information obtained by linking data from QregPV, a regional administrative health care database (Vega), the National Patient Register (NPR), the Prescribed Drug Register and the Cause of Death Register.

QregPV contains information about all primary care patients, irrespective of age or severity of disease, with a diagnosis of hypertension and CHD in VGR, including blood pressure, lipid levels, smoking, height and weight recorded during appointments and reported on a monthly basis. Starting in 2009, reporting to the register has been mandatory for all primary care units in VGR.

Vega covers all primary care in VGR since 2000. Data include diagnoses, dates and professional categories. NPR covers all hospital discharge diagnoses in Sweden since 1987, as well as outpatient appointments in specialised care since 2001. This study included NPR data since 1997, when the International Classification of Disease 10th revision (ICD-10) was adopted. The Prescribed Drug Register contains information about

For peer review only - http://bmjopen.bmj.com/site/about/guidelines.xhtml

all prescriptions filled since 1 July 2005. The Cause of Death Register consists of information about underlying and contributing causes of death since 1961.

All patients in QregPV with a diagnosis of CHD entered from 1 January 2010 to 31 December 2015 were included. Diagnoses were defined according to ICD-10: CHD I20-I25, AMI I21, stroke I61, I63 - I64, type 2 diabetes mellitus E10-14, heart failure I50 and atrial fibrillation and flutter I48. CVD was defined as acute myocardial infarction (AMI) or stroke.

Patients

Proceeding from the patient file, we created two cohorts:

- Study cohort (2015) – All patients with CHD in QregPV on 31 December 2015 (index date). Previous comorbidity until 31 December 2015 and prescriptions filled up to 120 days before the index date were collected from NPR, Vega and the Prescribed Drug Register.
- Risk estimation cohort (2011) – 52,042 patients with CHD in QregPV on 1 January 2011 (index date). Previous comorbidity until the index date and new events corresponding to diagnoses of AMI and stroke until 31 December 2015 were added from NPR and Vega. Information about deaths were retrieved from the Cause of Death Register.

For both cohorts, medical data were collected in accordance with the last observation carried forward (LOCF) method. We used LOCF in order to include as many patients as possible since we considered using the last measured value a better approach then imputation or further exclusion of patients due to missing values. The latest entry of each variable was used up to 900 days before the index date.

For peer review only - http://bmjopen.bmj.com/site/about/guidelines.xhtml

Risk estimation

The risk estimation cohort was used to create a model for the risk of recurrent CVD and all-cause mortality based on individual data for age, sex, diabetes mellitus, history of heart failure and/or atrial fibrillation, stroke or AMI during the previous year and treatment with acetylic salicylic acid. Statin treatment was not included since it is one of the factors we assess. Smoking and BMI were not included due to a large number of missing values. Blood pressure was not included due to missing data in 2010 and the J-curved association described in observational studies[16].

Prediction

We calculated the individual risk of CVD and death from all causes, for patients in the study cohort, using the risk prediction model. We performed two separate adjusted predictions and evaluated the effect of:

- lowering LDL-C to 1.8 mmol/L for all patients with a higher level
- adding atorvastatin 40/80 mg to patients with no or less potent statin therapy and LDL-C > 1.8 mmol/L.

For risk reduction associated with lowering of LDL-C, data from Cholesterol Treatment Trialists' (CTT) collaboration were used. Overall risk reduction for any major vascular event was 22% (rate ratio 0.78; 95% CI 0.76-0.80) per mmol/L reduction in LDL-C based on almost 170,000 individuals[3]. Risk reduction values for separate outcomes in the CTT study differed. Risk reduction per mmol/L LDL-C reduction for all-cause mortality was 0.91 based on results from another CTT collaboration study[17]. Risk reduction with intensified statin treatment proceeded from expected percentage LDL-C lowering related to type of statin and dosage based on studies concerning statin effect on LDL-C level[18, 19].

For peer review only - http://bmjopen.bmj.com/site/about/guidelines.xhtml

Statistical methods

Descriptive statistics are presented as arithmetic mean and standard deviation for continuous variables and with frequencies and percentages for categorical variables. A Cox regression model was applied to the risk estimation cohort. The variables included in the model were age, sex, diabetes mellitus, history of heart failure, history of atrial fibrillation, treatment with acetyl salicylic acid and stroke or AMI during the previous year. Age was modelled by means of a smoothing spline-function with four degrees of freedom since the effect of age was not assumed to be linear.

Time to event was right censored at death as end of follow up for total mortality and time to CVD. The time-at-risk for each patient was defined as the time before the first event over a period of five years.

The survival function and risk of having experienced an event over the past 5 years were calculated. Summing up individual risks yielded the total number of individuals predicted to have experienced an event over the past 5 years. Adjustments were made such that the cumulative hazard was recalculated based on an expected reduced risk for events related to lowering of LDL-C directly or expected lowering as a result of more intense statin treatment. The cumulative hazard of an event was reduced by 22% per mmol/L LDL-C lowering. This model did not take competing risks into account, assuming that all patients were alive after five years if they did not suffer an event.

The statistical analyses used R 3.4.0 and SAS version 9.4 (SAS Institute, Cary, NC).

Patient and public involvement

Patients or the public were not involved in the design, or conduct, or reporting, or dissemination plans of our research.

For peer review only

Results

Descriptive statistics for the study cohort are shown in

Table 1. There was a total of 57,341 patients, 37,120 of whom had information about LDL-C at baseline. Fewer than 20% (6,747/37,120) achieved the LDL-C target of ≤ 1.8 mmol/L. Among the non-controlled patients, with LDL-C > 1.8 mmol/L, an average LDL-C reduction of 1.2 mmol/L (40%) would be required to reach the LDL-C target. The subset of patients with AMI or stroke in the previous year had a mean age of 74.2 (10.5); 78% received statin treatment and 25% reached the LDL-C target.

Table 1. Characteristics of patients in the study cohort.

**Variable**	**All patients (n = 37,120)**	**LDL-C <= 1.8 mmol/L (n= 6,747)**	**LDL-C > 1.8 mmol/L (n= 30,373)**
Age	73.0 (10.1)	73.2 (9.9)	73.0 (10.1)
Sex (female)	13,585 (36.6%)	1,897 (28.1%)	11,688 (38.5%)
Smoking	4,221 (12.7%)	755 (12.3%)	3,466 (12.7%)
SBP (mmHg)	132.8 (16.1)	130.6 (15.6)	133.3 (16.2)
DBP (mmHg)	75.3 (10.7)	73.9 (10.7)	75.6 (10.7)
Total cholesterol (mmol/L)	4.5 (1.2)	3.4 (0.7)	4.8 (1.1)
LDL-C (mmol/L)	2.7 (1.0)	1.5 (0.3)	3.0 (0.9)
Triglyceride (mmol/L)	1.6 (0.9)	1.5 (1.0)	1.6 (0.9)
Hypertension	30,869 (83.2%)	5,649 (83.7%)	25,220 (83.0%)
Diabetes	12,544 (33.8%)	3,116 (46.2%)	9,428 (31.0%)
CHD	37,120 (100.0%)	6,747 (100.0%)	30,373 (100.0%)

For peer review only - http://bmjopen.bmj.com/site/about/guidelines.xhtml

Variable	All patients (n = 37,120)	LDL-C <= 1.8 mmol/L (n= 6,747)	LDL-C > 1.8 mmol/L (n= 30,373)
AMI	16,742 (45.1%)	3,740 (55.4%)	13,002 (42.8%)
AMI past year	1,544 (4.2%)	441 (6.5%)	1,103 (3.6%)
Stroke	4,452 (12.0%)	921 (13.7%)	3,531 (11.6%)
Stroke past year	1,292 (3.5%)	266 (3.9%)	1,026 (3.4%)
CVD	19,183 (51.7%)	4,177 (61.9%)	15,006 (49.4%)
Heart failure	8,888 (23.9%)	1,951 (28.9%)	6,937 (22.8%)
Atrial fibrillation	8,257 (22.2%)	1,807 (26.8%)	6,450 (21.2%)
Dementia	1,363 (3.7%)	224 (3.3%)	1,139 (3.8%)
ASA	23,431 (63.1%)	4,426 (65.6%)	19,005 (62.6%)
Statin	25,160 (67.8%)	5,735 (85.0%)	19,425 (64.0%)
Simvastatin	12,820 (34.5%)	2,810 (41.6%)	10,010 (33.0%)
Pravastatin	286 (0.8%)	20 (0.3%)	266 (0.9%)
Atorvastatin	11,424 (30.8%)	2,723 (40.4%)	8,701 (28.6%)
Rosuvastatin	998 (2.7%)	235 (3.5%)	763 (2.5%)
Other lipid lowering drugs	1,231 (3.3%)	227 (3.4%)	1,004 (3.3%)
Ezetimib	953 (2.6%)	175 (2.6%)	778 (2.6%)

Mean (SD) and frequencies (%). Abbreviations: SBP: Systolic blood pressure; DBP: Diastolic blood pressure; LDL-C: Low density lipoprotein cholesterol; CHD: coronary heart disease; AMI: acute myocardial infarction; CVD: cardiovascular disease; ASA: acetylic salicylic acid.

For peer review only - http://bmjopen.bmj.com/site/about/guidelines.xhtml

Statin treatment was most prevalent for age 50-75, approximately 70% of whom filled a prescription, see Figure 1. Prescriptions filled for statins diminish rapidly after the age of 80.

Figure 1.

LDL-C for uncontrolled patients was predicted to decrease from a mean of 3.0 to 2.2 mmol/L if all patients received atorvastatin 40 mg and to 2.0 mmol/L with atorvastatin 80 mg. For all patients in the study cohort the corresponding levels were 2.7 to 2.1 and 1.9 mmol/L, see LDL-C and predicted LDL-C distribution in Figure 2.

Figure 2.

Total predicted CVD events for 5 years in the study cohort was 9,209 (24.8%), see Figure 3. A decrease of LDL-C to the target (≤ 1.8 mmol/l) or intensified statin treatment (all patients with less efficient therapy receive atorvastatin 80 mg) would result in a reduction of expected CVD events for 5 years by 1,632 and 1,308. This would result in a decreased event risk of 18% and 14%. Including only the patients with LDL-C > 1.8, the corresponding reductions were 22% and 18%. All-cause mortality for 5 years declined by 6.4 % (534/8,344) when LDL-C was lowered to ≤ 1.8 mmol/L.

Figure 3.

Sensitivity analyses by adding variables showed that the model was robust (supplementary table I). When BMI and smoking status were included, the number of patients in the risk estimation cohort was reduced, from 52,042 to 9,254. The proportion of patients who were predicted to suffer a CVD event was decreased from 25% to 22% but the relative

For peer review only - http://bmjopen.bmj.com/site/about/guidelines.xhtml
For peer review only

reduction in number of events was the same. The results were similar without including BMI and smoking status in the model. The same was true if the risk estimation model was based on the 16,577 patients with LDL-C (although LDL-C was not in the model). When systolic blood pressure was included in the model, the risk estimation cohort was decreased to 35,989 and the outcomes were relatively unchanged. Inclusion of statin treatment (0/1) in the model did not lead to any changes in the results.

The proportion of patients predicted to experience a CVD event was 24.6 % (2,937/11,960) among non-statin users and 24.9 % (6,271/25,160) among statin users, see Figure 4. If all patients were prescribed 80 mg atorvastatin, the predicted proportion to experience a CVD event was reduced by 32.9 % (967) among non-statin users and 5.4 % (339) among statin users. The patients with statin treatment were younger (72.3 vs 74.5) and consisted of more men (68% vs 54%), had a diabetes diagnosis to a larger extent (36% vs 29%) and more often had a stroke or AMI during the past year (4-5% vs 2.5%) (supplementary table II).

Figure 4.

Other lipid-lowering drugs were rarely used, 953/37,120 (2.6%) only 442 of whom were not also on statin. Patients receiving other lipid-lowering drugs had slightly higher LDL-C than the rest of the study population.

Discussion

Summary

This study shows that the number of CVD events could be substantially reduced if patients with no/low-dose statin were given atorvastatin 80 mg and even more so if all patients reached the LDL-C target. 1/3 of our study population had no statin treatment and

For peer review only

For peer review only - http://bmjopen.bmj.com/site/about/guidelines.xhtml

fewer than 1/5 reached the recommended LDL-C target of 1.8 mmol/L. The largest gain would result from initiating treatment in patients who are not receiving statin.

Strengths and limitations

The main strength of our study was the large number of primary care patients with CHD. Previous studies were often based on a post-MI population from secondary care registers. Another strength is the completeness of data concerning comorbidities and filled prescriptions. We based our risk model calculations on the same regional population as the predictions.

Information about LDL-C level was missing for many patients in the study cohort. These patients were older, diagnosed more often with atrial fibrillation and heart failure, less with diabetes and not as likely to be receiving statin treatment. Inclusion of them would probably have increased the predicted number of saved events. BMI, smoking and blood pressure were not included in the model due to the quantity of missing values in the risk estimation cohort. Sensitivity analyses showed that the results remained approximately the same without these variables. We assumed that all patients benefit proportionally from better adherence and improved treatment. The patients with appropriate treatment might differ from those without as can also be a problem in RCTs. It has been shown that compliance per se (placebo or active treatment) is associated with lower mortality [20]. In our study, patients with no statin treatment could either have no prescription by a physician or be non-compliant. We were not able to adjust for this difference but since our aim was not to estimate causal effects but to get an estimation of the effects of a changed treatment pattern, we consider our results still valid.

We did not take competing risks into account and assumed that no patient had died from other causes during the follow-up period. Patients in our risk estimation cohort who died before a potential event were right censored and 9.2% died from other causes without a

For peer review only - http://bmjopen.bmj.com/site/about/guidelines.xhtml

previous event. Our model appears to slightly overestimate the number of CVD events, but the percentage decrease is not necessarily affected.

Comparison with existing literature

A recent Swedish study described short-term adherence to statins and potential effects of improved treatment among 5,904 post-MI patients[21]. The study found an estimated 805-2,262 CVD events in over 10 years among those who did not reach LDL-C target and predicted that a reduction of LDL-C to 1.8 mmol/L would lower the number by 132-343 (15-16%). The patients in that study were younger, which might explain the smaller predicted reduction in events.

LDL-C is monitored one year after AMI in the Swedish national quality register (SEPHIA). In 2015, mean LDL-C was 1.99 mmol/L and 51% had LDL-C < 1.8 mmol/L[22]. Short-term adherence to lipid lowering treatment after AMI is gradually improving in Sweden[15]. A Norwegian study of 42,707 patients discharged from hospital after AMI in 2009-2013 reported that 84% were being treated with statins one year later[8]. Mean LDL-C was 2.7 mmol/L in our study cohort and only 18% had LDL-C ≤ 1.8 mmol/L also including those younger than 75 as those in SEPHIA. This difference between improved treatment in the short term and our finding of poor control in the long term may be due to poorer adherence to lipid-lowering treatment over time and the fact that new guidelines are applied to patients with a recent AMI event. Poor long-term adherence has been demonstrated earlier with discontinuation rates of 67% over 5 years[23]. The probability of persistence has increased over the years but remains low already a few years after initiation of treatment[24]. Over the past few years, statin treatment has proven to be safe and effective among the aged as well, but the proportion of patients in our study receiving it diminishes after the age of 75[2]. The importance of

For peer review only

For peer review only - http://bmjopen.bmj.com/site/about/guidelines.xhtml

continued statin treatment is further stressed by the fact that LDL-C remains an important risk factor for CVD even at high age as opposed to other traditional risk factors [1].

Our cohort consists of primary care patients with a CHD diagnosis. It has been shown that patients in primary care with known CVD from diagnoses in hospital but without a registered one in primary care receive less statin treatment[25]. In light of these results, our study presumably underestimates the potential event reduction by means of improved treatment.

The reasons for non-adherence to guidelines may depend on the physician, patient or organisational factors. A recent Swedish study showed that patients with concomitant use of other cardio-protective medications or smoking had higher adherence to refill and lipid-lowering treatment[26]. Two Danish studies found adherence to statin treatment to be lower among patients with a more risk-seeking attitude and physicians who did not assess treatment after initiation[27, 28]. The reasons that physicians do not adhere to lipid-lowering guidelines may be the same as for the blood pressure target: acceptance of higher values than recommended, competing medical problems, etc.[29]. Our results suggest a large potential for improvement of secondary prevention in primary care by improved statin treatment and especially initiate treatment in non-statin users.

Implications for research and/or practice

The treatment gap for lipid-lowering therapy in a primary care population with established CHD was large, representing a significant potential to reduce the number of events if treatment guidelines were to be followed to a greater extent.

For peer review only

Funding

The authors disclosed receipt of the following financial support for the research, authorship, and/or publication of this article: This work was supported by Närhälsan R&D Health Care, R&D Centre Gothenburg and Södra Bohuslän for postgraduate studies. This work was also supported by grants from the Swedish government under the Agreement concerning Research and Education of Doctors [grant number ALFGBG-717211], the Swedish Heart and Lung Foundation [grant number 2018-0366] and the Swedish Research Council [grant number 2018-02527].

Declaration of Conflicting Interests

SAE is employed by AstraZeneca since September 2019 but was affiliated to University of Gothenburg during the main part of the study.

Author contributions

HÖ, SB, SF, PH, KM, AR and SAE contributed to the conception and design of the work. All authors contributed to the acquisition, analysis, or interpretation of data for the work. HÖ drafted the manuscript. All authors critically revised the manuscript. All authors gave final approval and agree to be accountable for all aspects of work ensuring integrity and accuracy.

Data sharing statement

No additional unpublished data are available.

For peer review only

For peer review only - http://bmjopen.bmj.com/site/about/guidelines.xhtml

References

1. Lind L, Sundstrom J, Arnlov J, Lampa E. Impact of Aging on the Strength of Cardiovascular Risk Factors: A Longitudinal Study Over 40 Years. J Am Heart Assoc. 2018;7(1).

2. Cholesterol Treatment Trialists C, Armitage J, Baigent C, Barnes E, Betteridge D. Efficacy and safety of statin therapy in older people: a meta-analysis of individual participant data from 28 randomised controlled trials. Lancet. 2019;393(10170):407-15.

3. Cholesterol Treatment Trialists C, Baigent C, Blackwell L, Emberson J, Holland LE, Reith C, Bhala N, Peto R, Barnes EH, Keech A, Simes J, Collins R. Efficacy and safety of more intensive lowering of LDL cholesterol: a meta-analysis of data from 170,000 participants in 26 randomised trials. Lancet. 2010;376(9753):1670-81.

4. Ference BA, Ginsberg HN, Graham I, Ray KK, Packard CJ, Bruckert E, Hegele RA, Krauss RM, Raal FJ, Schunkert H, Watts GF, Boren J, Fazio S, Horton JD, Masana L, Nicholls SJ, Nordestgaard BG, van de Sluis B, Taskinen MR, Tokgozoglu L, Landmesser U, Laufs U, Wiklund O, Stock JK, Chapman MJ, Catapano AL. Low-density lipoproteins cause atherosclerotic cardiovascular disease. 1. Evidence from genetic, epidemiologic, and clinical studies. A consensus statement from the European Atherosclerosis Society Consensus Panel. Eur Heart J. 2017;38(32):2459-72.

5. Statistik om dödsorsaker 2017 [Cause of Death statistics 2017]. https://www.socialstyrelsen.se/Lists/Artikelkatalog/Attachments/21101/2018-10-17.pdf: Social Board of Health and Welfare, 2018.

6. Rodriguez F, Maron DJ, Knowles JW, Virani SS, Lin S, Heidenreich PA. Association of Statin Adherence With Mortality in Patients With Atherosclerotic Cardiovascular Disease. JAMA Cardiol. 2019;4(3):206-13.

7. Banach M, Stulc T, Dent R, Toth PP. Statin non-adherence and residual cardiovascular risk: There is need for substantial improvement. Int J Cardiol. 2016;225:184-96.

For peer review only

For peer review only - http://bmjopen.bmj.com/site/about/guidelines.xhtml

8. Halvorsen S, Jortveit J, Hasvold P, Thuresson M, Oie E. Initiation of and long-term adherence to secondary preventive drugs after acute myocardial infarction. BMC Cardiovasc Disord. 2016;16:115.

9. Ofori-Asenso R, Jakhu A, Zomer E, Curtis AJ, Korhonen MJ, Nelson M, Gambhir M, Tonkin A, Liew D, Zoungas S. Adherence and Persistence Among Statin Users Aged 65 Years and Over: A Systematic Review and Meta-analysis. J Gerontol A Biol Sci Med Sci. 2018;73(6):813-9.

10. Tramacere I, Boncoraglio GB, Banzi R, Del Giovane C, Kwag KH, Squizzato A, Moja L. Comparison of statins for secondary prevention in patients with ischemic stroke or transient ischemic attack: a systematic review and network meta-analysis. BMC Med. 2019;17(1):67.

11. Tobert JA, Newman CB. Statin tolerability: In defence of placebo-controlled trials. Eur J Prev Cardiol. 2016;23(8):891-6.

12. Kruger K, Leppkes N, Gehrke-Beck S, Herrmann W, Algharably EA, Kreutz R, Heintze C, Filler I. Improving long-term adherence to statin therapy: a qualitative study of GPs' experiences in primary care. Br J Gen Pract. 2018;68(671):e401-e7.

13. To prevent atherosclerotic cardiovascular disease with drugs - treatment recommendations. Stockholm. Sweden. Medical Product Agency; updated 2014; Available at: https://lakemedelsverket.se/upload/halso-och-sjukvard/behandlingsrekommendationer/Att_forebygga_aterosklerotisk_hjart-karlsjukdom_med%20_lakemedel_behandlingsrekommendation.pdf (accessed 7/12 2018).

14. Authors/Task Force M, Piepoli MF, Hoes AW, Agewall S, Albus C, Brotons C, Catapano AL, Cooney MT, Corra U, Cosyns B, Deaton C, Graham I, Hall MS, Hobbs FD, Lochen ML, Lollgen H, Marques-Vidal P, Perk J, Prescott E, Redon J, Richter DJ, Sattar N, Smulders Y, Tiberi M, van der Worp HB, van Dis I, Verschuren WM, Additional Contributor: Simone B, Document R, De Backer G, Roffi M, Aboyans V, Bachl N, Bueno H, Carerj S, Cho L, Cox J, De Sutter J, Egidi G, Fisher M, Fitzsimons D, Franco OH, Guenoun M, Jennings C, Jug B, Kirchhof P, Kotseva K, Lip GY, Mach F, Mancia G, Bermudo FM, Mezzani A, Niessner A, Ponikowski P, Rauch B, Ryden L, Stauder A, Turc G, Wiklund O, Windecker

For peer review only

For peer review only - http://bmjopen.bmj.com/site/about/guidelines.xhtml

S, Zamorano JL. 2016 European Guidelines on cardiovascular disease prevention in clinical practice: The Sixth Joint Task Force of the European Society of Cardiology and Other Societies on Cardiovascular Disease Prevention in Clinical Practice (constituted by representatives of 10 societies and by invited experts): Developed with the special contribution of the European Association for Cardiovascular Prevention & Rehabilitation (EACPR). Eur J Prev Cardiol. 2016;23(11):NP1-NP96. Epub 2016/06/30.

15. Swedeheart. SWEDEHEART Annual Report 2017. Stockholm: 2017.

16. Adamsson Eryd S, Gudbjornsdottir S, Manhem K, Rosengren A, Svensson AM, Miftaraj M, Franzen S, Bjorck S. Blood pressure and complications in individuals with type 2 diabetes and no previous cardiovascular disease: national population based cohort study. BMJ. 2016;354:i4070. Epub 2016/08/06.

17. Cholesterol Treatment Trialists C, Fulcher J, O'Connell R, Voysey M, Emberson J, Blackwell L, Mihaylova B, Simes J, Collins R, Kirby A, Colhoun H, Braunwald E, La Rosa J, Pedersen TR, Tonkin A, Davis B, Sleight P, Franzosi MG, Baigent C, Keech A. Efficacy and safety of LDL-lowering therapy among men and women: meta-analysis of individual data from 174,000 participants in 27 randomised trials. Lancet. 2015;385(9976):1397-405.

18. Law MR, Wald NJ, Rudnicka AR. Quantifying effect of statins on low density lipoprotein cholesterol, ischaemic heart disease, and stroke: systematic review and meta-analysis. BMJ. 2003;326(7404):1423.

19. NICE. NICE Guidance - Cardiovascular disease: risk assessment and reduction, including lipid modification. London: The National Institute for Health and Care Excellence (NICE); 2014 updated 2016; Available at: https://www.nice.org.uk/guidance/cg181/chapter/appendix-a-grouping-of-statins (accessed 14/11 2018).

20. Coronary Drug Project Research G. Influence of adherence to treatment and response of cholesterol on mortality in the coronary drug project. N Engl J Med. 1980;303(18):1038-41.

For peer review only

For peer review only - http://bmjopen.bmj.com/site/about/guidelines.xhtml

21. Journath G, Hambraeus K, Hagstrom E, Pettersson B, Lothgren M. Predicted impact of lipid lowering therapy on cardiovascular and economic outcomes of Swedish atherosclerotic cardiovascular disease guideline. BMC Cardiovasc Disord. 2017;17(1):224.

22. SWEDEHEART. SWEDEHEART Annual report 2015. Stockholm: 2015.

23. Ofori-Asenso R, Ilomaki J, Tacey M, Zomer E, Curtis AJ, Bell JS, Zoungas S, Liew D. Patterns of statin use and long-term adherence and persistence among older adults with diabetes. J Diabetes. 2018;10(9):699-707.

24. Chen ST, Huang ST, Shau WY, Lai CL, Li JZ, Fung S, Tse VC, Lai MS. Long-term statin adherence in patients after hospital discharge for new onset of atherosclerotic cardiovascular disease: a population-based study of real world prescriptions in Taiwan. BMC Cardiovasc Disord. 2019;19(1):62.

25. Dahlgren C, Geary L, Hasselstrom J, Rehnberg C, Schenck-Gustafsson K, Wandell P, Euler MV. Recording a diagnosis of stroke, transient ischaemic attack or myocardial infarction in primary healthcare and the association with dispensation of secondary preventive medication: a registry-based prospective cohort study. BMJ Open. 2017;7(9):e015723.

26. Karlsson SA, Hero C, Eliasson B, Franzen S, Svensson AM, Miftaraj M, Gudbjornsdottir S, Eeg-Olofsson K, Andersson Sundell K. Refill adherence and persistence to lipid-lowering medicines in patients with type 2 diabetes: A nation-wide register-based study. Pharmacoepidemiol Drug Saf. 2017;26(10):1220-32.

27. Barfoed BL, Paulsen MS, Christensen PM, Halvorsen PA, Jarbol DE, Larsen ML, Munch MR, Sondergaard J, Nielsen JB. Associations between patients' adherence and GPs' attitudes towards risk, statin therapy and management of non-adherence--a survey and register-based study. Fam Pract. 2016;33(2):140-7.

28. Barfoed BL, Paulsen MS, Christensen PM, Halvorsen PA, Kjaer T, Larsen ML, Larsen PV, Nielsen JB, Sondergaard J, Jarbol DE. Associations between patients' risk attitude and their

For peer review only

For peer review only - http://bmjopen.bmj.com/site/about/guidelines.xhtml

adherence to statin treatment - a population based questionnaire and register study. BMC Fam Pract. 2016;17:28.

29. Oliveria SA, Lapuerta P, McCarthy BD, L'Italien GJ, Berlowitz DR, Asch SM. Physician-related barriers to the effective management of uncontrolled hypertension. Arch Intern Med. 2002;162(4):413-20.

For peer review only

For peer review only - http://bmjopen.bmj.com/site/about/guidelines.xhtml

Legends

Figure 1. Statin treatment by age. Included ages with more than 100 observations. The number of patients below the age of 50 are very few (603/37,120) as are the patients over the age of 90 (925/37,120).

Figure 2. LDL-C distribution in all patients in the study cohort with current treatment and adjusted treatment defined as if all patients received atorvastatin 80 mg (if less intense treatment before and LDL-C > 1.8 mmol/L).

Figure 3. Predicted number of events in the study cohort (37,120 patients) with registered levels of LDL-C and current treatment as well as when modelling a reduction in LDL-C to 1.8 mmol/L for all patients with higher LDL-C or an intensified statin lowering treatment with 80 mg atorvastatin for those with a less efficient treatment.

Figure 4. Predicted number of events in the study cohort with registered levels of LDL-C and current treatment separated in patients with and without current statin treatment and the predicted number of events when applying a more intense statin treatment to all patients with a less efficient treatment.

For peer review only

For peer review only - http://bmjopen.bmj.com/site/about/guidelines.xhtml

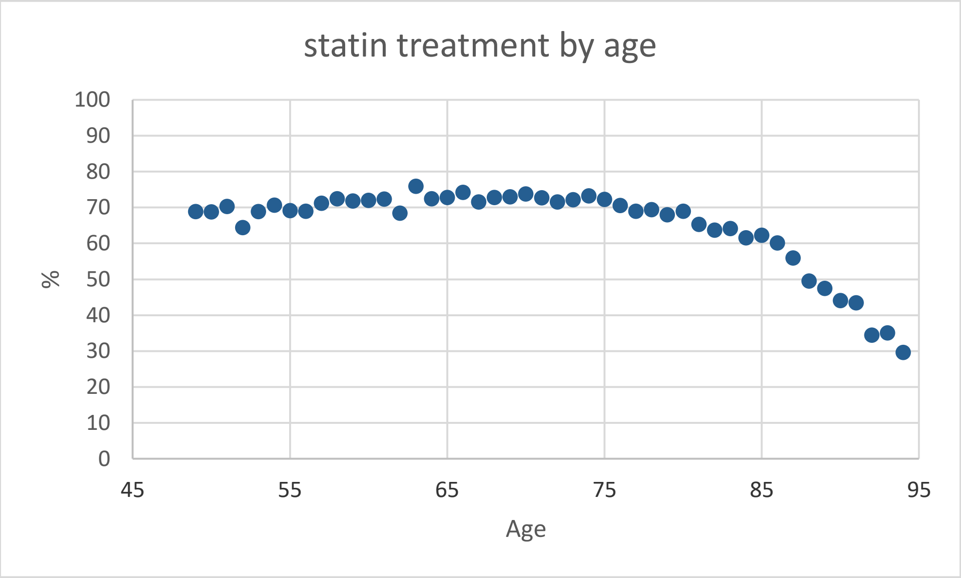

peer review only

1 (1)

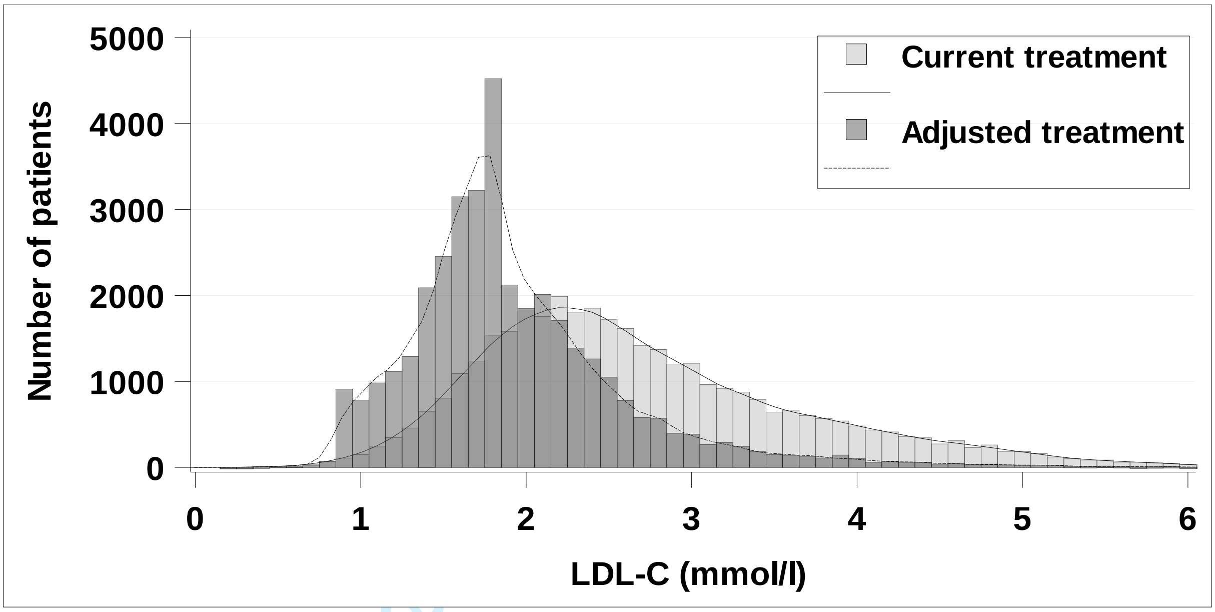
Number of patients
5000
4000
3000
2000
1000
0
Current treatment
Adjusted treatment
0
1
2
3
4
5
6
LDL-C (mmol/l)

1 (**1**)

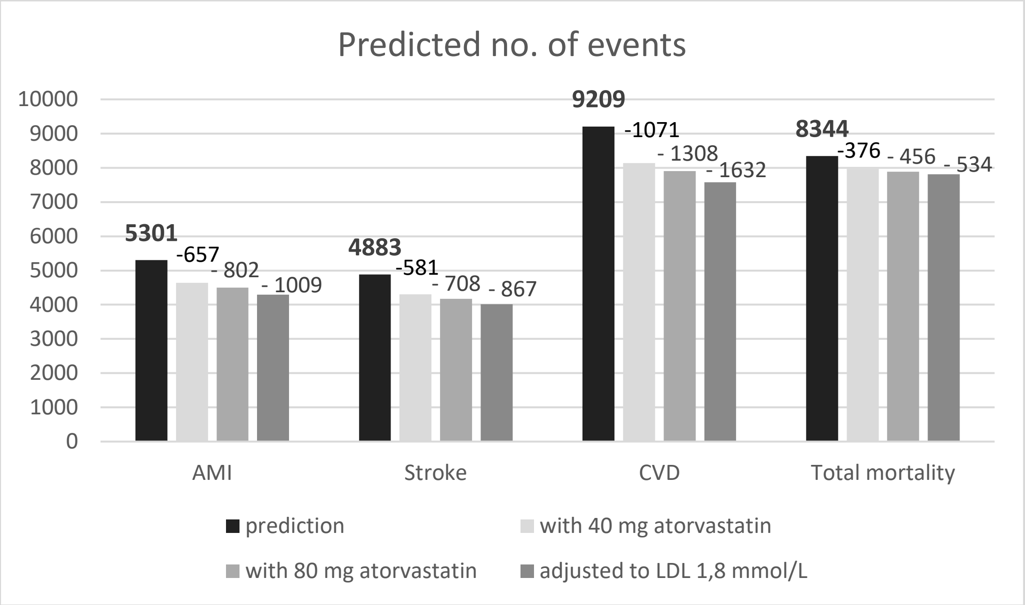
Predicted no. of events
10000
9000
8000
7000
6000
5000
4000
3000
2000
1000
0
5301
-657
- 802
- 1009
4883
-581
- 708
- 867
9209
-1071
- 1308
- 1632
8344
-376
- 456
- 534
AMI
Stroke
CVD
Total mortality
prediction
with 40 mg atorvastatin
with 80 mg atorvastatin
adjusted to LDL 1,8 mmol/L

1 (1)

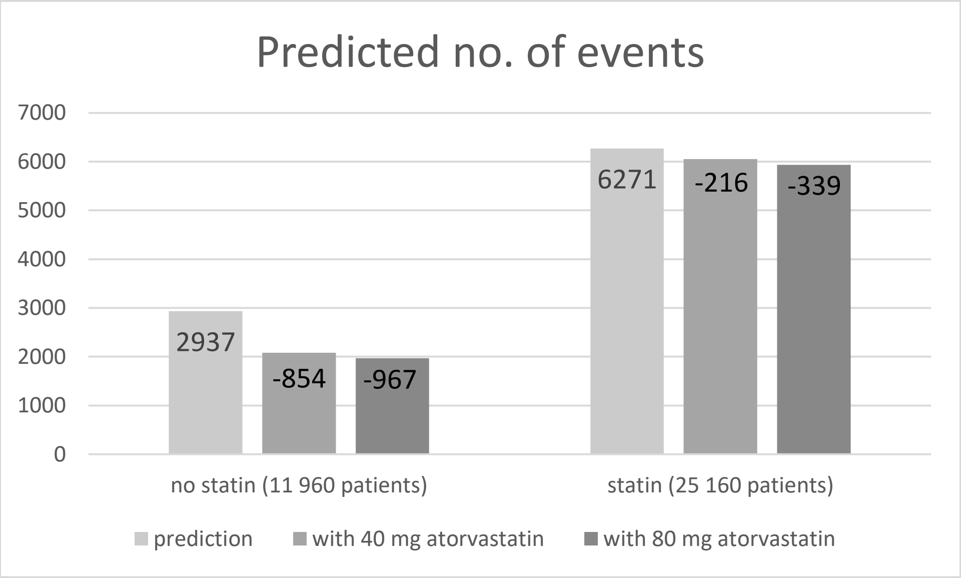

1 (1)

For peer review only - http://bmjopen.bmj.com/site/about/guidelines.xhtml

Supplementary information – Adherence to lipid-lowering guidelines for secondary prevention and potential reduction in CVD events in Swedish primary care – a cross-sectional study. Ödesjö et al

Table I. Sensitivity analyses

	Percentage estimated events	Percentage estimated events with LDL-C < 1.8 mmol/L	Reduction in number of events
**Model presented in the manuscript**	24.8%	20.4%	17.7%
**Data with BMI/smoking[1]**	22.1%	18.3%	17.1%
**Data with LDL-C[2]**	22.2%	18.3%	17.6%
**With SBP in model[3]**	23.9%	19.7%	17.7%
**With statin in model[4]**	24.6%	20.2%	18.0%

[1] Calculations based on the patients in the risk and study cohorts with information on BMI and smoking. The same risk model was applied as in the presented material as well as a model including BMI and smoking. Both models resulted in similar percentages.

[2] Calculations based on the patients in the risk and study cohorts with information on LDL-C. The same risk model applied as in the presented material.

[3] Calculations based on the same risk and study cohorts but with SBP added to the model, patients without information on SBP were thereby not included.

[4] Calculations based on the same risk and study cohorts but with statin added to the model.

For peer review only

Table II. Characteristics of patients in the study cohort divided by statin treatment or not.

Variable	No statin treatment (n= 11960)	Statin treatment (n= 25160)
Age	74.5 (11.0)	72.3 (9.5)
Sex (female)	5,529 (46.2%)	8,056 (32.0%)
Smoking	1,300 (12.5%)	2,921 (12.7%)
SBP (mmHg)	134.3 (17.0)	132.1 (15.6)
DBP (mmHg)	76.1 (11.2)	74.9 (10.4)
Total cholesterol (mmol/L)	5.2 (1.3)	4.2 (1.0)
LDL-C (mmol/L)	3.3 (1.1)	2.4 (0.8)
Triglyceride (mmol/L)	1.6 (0.9)	1.5 (0.9)
Hypertension	9,858 (82.4%)	21,011 (83.5%)
Diabetes	3488 (29.2%)	9056 (36.0%)
CHD	11,960 (100.0%)	25,160 (100.0%)
AMI	4,001 (33.5%)	12,741 (50.6%)
AMI past year	285 (2.4%)	1,259 (5.0%)
Stroke	1,315 (11.0%)	3,137 (12.5%)
Stroke past year	323 (2.7%)	969 (3.9%)
CVD	4,798 (40.1%)	14,385 (57.2%)

For peer review only

Heart failure	3,070 (25.7%)	5,818 (23.1%)
Atrial fibrillation	2,853 (23.9%)	5,404 (21.5%)
Dementia	589 (4.9%)	774 (3.1%)
ASA	5402 (45.2%)	18029 (71.7%)
Statin		25160 (100.0%)
Simvastatin		12820 (51.0%)
Pravastatin		286 (1.1%)
Atorvastatin		11424 (45.4%)
Rosuvastatin		998 (4.0%)
Other lipid lowering drugs	442 (3.7%)	789 (3.1%)
Ezetimib	277 (2.3%)	676 (2.7%)

Mean (SD) and frequencies (%). Abbreviations: SBP: Systolic blood pressure; DBP: Diastolic blood pressure; LDL-C: Low density lipoprotein cholesterol; CHD: coronary heart disease; AMI: acute myocardial infarction; CVD: cardiovascular disease; ASA: acetylic salicylic acid.

For peer review only

STROBE 2007 (v4) Statement—Checklist of items that should be included in reports of *cross-sectional studies*

Section/Topic	Item #	Recommendation	Reported on page #
**Title and abstract**	1	(*a*) Indicate the study's design with a commonly used term in the title or the abstract	Yes
		(*b*) Provide in the abstract an informative and balanced summary of what was done and what was found	Yes
**Introduction**			
Background/rationale	2	Explain the scientific background and rationale for the investigation being reported	Yes
Objectives	3	State specific objectives, including any prespecified hypotheses	Yes
**Methods**			
Study design	4	Present key elements of study design early in the paper	Yes
Setting	5	Describe the setting, locations, and relevant dates, including periods of recruitment, exposure, follow-up, and data collection	Yes
Participants	6	(*a*) Give the eligibility criteria, and the sources and methods of selection of participants	Yes
Variables	7	Clearly define all outcomes, exposures, predictors, potential confounders, and effect modifiers. Give diagnostic criteria, if applicable	Yes
Data sources/ measurement	8*	For each variable of interest, give sources of data and details of methods of assessment (measurement). Describe comparability of assessment methods if there is more than one group	Yes
Bias	9	Describe any efforts to address potential sources of bias	*Yes*
Study size	10	Explain how the study size was arrived at	
Quantitative variables	11	Explain how quantitative variables were handled in the analyses. If applicable, describe which groupings were chosen and why	Yes
Statistical methods	12	(*a*) Describe all statistical methods, including those used to control for confounding	Yes
		(*b*) Describe any methods used to examine subgroups and interactions	NA
		(*c*) Explain how missing data were addressed	Yes
		(*d*) If applicable, describe analytical methods taking account of sampling strategy	NA
		(*e*) Describe any sensitivity analyses	Yes
**Results**			

For peer review only

Participants	13*	(a) Report numbers of individuals at each stage of study—eg numbers potentially eligible, examined for eligibility, confirmed eligible, included in the study, completing follow-up, and analysed	Yes
		(b) Give reasons for non-participation at each stage	Yes
		(c) Consider use of a flow diagram	
Descriptive data	14*	(a) Give characteristics of study participants (eg demographic, clinical, social) and information on exposures and potential confounders	Yes
		(b) Indicate number of participants with missing data for each variable of interest	Yes
Outcome data	15*	Report numbers of outcome events or summary measures	Yes
Main results	16	(*a*) Give unadjusted estimates and, if applicable, confounder-adjusted estimates and their precision (eg, 95% confidence interval). Make clear which confounders were adjusted for and why they were included	NA
		(*b*) Report category boundaries when continuous variables were categorized	NA
		(*c*) If relevant, consider translating estimates of relative risk into absolute risk for a meaningful time period	
Other analyses	17	Report other analyses done—eg analyses of subgroups and interactions, and sensitivity analyses	Yes
**Discussion**			
Key results	18	Summarise key results with reference to study objectives	Yes
Limitations	19	Discuss limitations of the study, taking into account sources of potential bias or imprecision. Discuss both direction and magnitude of any potential bias	Yes
Interpretation	20	Give a cautious overall interpretation of results considering objectives, limitations, multiplicity of analyses, results from similar studies, and other relevant evidence	Yes
Generalisability	21	Discuss the generalisability (external validity) of the study results	Yes
**Other information**			
Funding	22	Give the source of funding and the role of the funders for the present study and, if applicable, for the original study on which the present article is based	Yes

*Give information separately for cases and controls in case-control studies and, if applicable, for exposed and unexposed groups in cohort and cross-sectional studies.

Note: An Explanation and Elaboration article discusses each checklist item and gives methodological background and published examples of transparent reporting. The STROBE checklist is best used in conjunction with this article (freely available on the Web sites of PLoS Medicine at http://www.plosmedicine.org/, Annals of Internal Medicine at http://www.annals.org/, and Epidemiology at http://www.epidem.com/). Information on the STROBE Initiative is available at www.strobe-statement.org.

For peer review only

BMJ Open

Adherence to lipid-lowering guidelines for secondary prevention and potential reduction in CVD events in Swedish primary care – a cross-sectional study

Journal:	*BMJ Open*
Manuscript ID	bmjopen-2020-036920.R2
Article Type:	Original research
Date Submitted by the Author:	29-Aug-2020
Complete List of Authors:	Ödesjö, Helena; University of Gothenburg Sahlgrenska Academy, Primary Health Care, School of Public Health and Community Medicine, Institute of Medicine<br>Björck, Staffan; Centre of Registers Västra Götaland<br>Franzén, Stefan; Centre of Registers Västra Götaland<br>Hjerpe, Per; University of Gothenburg Sahlgrenska Academy, Primary Health Care, School of Public Health and Community Medicine, Institute of Medicine<br>Manhem, Karin; University of Gothenburg Department of Molecular and Clinical Medicine<br>Rosengren, Annika; University of Gothenburg Department of Molecular and Clinical Medicine<br>Thorn, Jörgen; University of Gothenburg Sahlgrenska Academy, Primary Health Care, School of Public Health and Community Medicine, Institute of Medicine<br>Adamsson Eryd, Samuel; University of Gothenburg Department of Molecular and Clinical Medicine
**Primary Subject Heading**:	General practice / Family practice
Secondary Subject Heading:	Cardiovascular medicine, General practice / Family practice
Keywords:	Coronary heart disease < CARDIOLOGY, PRIMARY CARE, Cardiac Epidemiology < CARDIOLOGY, Lipid disorders < DIABETES & ENDOCRINOLOGY

SCHOLARONE™
Manuscripts

BMJ

I, the Submitting Author has the right to grant and does grant on behalf of all authors of the Work (as defined in the below author licence), an exclusive licence and/or a non-exclusive licence for contributions from authors who are: i) UK Crown employees; ii) where BMJ has agreed a CC-BY licence shall apply, and/or iii) in accordance with the terms applicable for US Federal Government officers or employees acting as part of their official duties; on a worldwide, perpetual, irrevocable, royalty-free basis to BMJ Publishing Group Ltd ("BMJ") its licensees and where the relevant Journal is co-owned by BMJ to the co-owners of the Journal, to publish the Work in this journal and any other BMJ products and to exploit all rights, as set out in our licence.

The Submitting Author accepts and understands that any supply made under these terms is made by BMJ to the Submitting Author unless you are acting as an employee on behalf of your employer or a postgraduate student of an affiliated institution which is paying any applicable article publishing charge ("APC") for Open Access articles. Where the Submitting Author wishes to make the Work available on an Open Access basis (and intends to pay the relevant APC), the terms of reuse of such Open Access shall be governed by a Creative Commons licence – details of these licences and which Creative Commons licence will apply to this Work are set out in our licence referred to above.

Other than as permitted in any relevant BMJ Author's Self Archiving Policies, I confirm this Work has not been accepted for publication elsewhere, is not being considered for publication elsewhere and does not duplicate material already published. I confirm all authors consent to publication of this Work and authorise the granting of this licence.

For peer review only

Adherence to lipid-lowering guidelines for secondary prevention and potential reduction in CVD events in Swedish primary care – a cross-sectional study

H. Ödesjö[ab], S. Björck[d], S. Franzén[d], P. Hjerpe[ae], K. Manhem[c], A. Rosengren[c], J. Thorn[a], S. Adamsson Eryd[c]

[a]Primary Health Care, School of Public Health and Community Medicine, Institute of Medicine, Sahlgrenska Academy, University of Gothenburg, SE 405 30 Gothenburg, Sweden

[b]Primary Health Care, Region Västra Götaland, Närhälsan Tjörn Vårdcentral, Syster Ebbas väg, SE-471 94 Kållekärr, Sweden

[c]Department of Molecular and Clinical Medicine, Institute of Medicine, Sahlgrenska Academy, University of Gothenburg and Sahlgrenska University Hospital, Östra Hospital, Diagnosvägen 11, SE 416 50 Gothenburg, Sweden

[d]Centre of Registers Västra Götaland, Medicinaregatan 18G, SE 413 45 Gothenburg, Sweden

[e]R&D Centre Skaraborg Primary Care, Skövde, Regionens hus, SE54130 Skövde, Sweden

Contact information:

Helena Ödesjö, MD, PhD student: helena.odesjo@vgregion.se, +46 73 561 78 23

(corresponding author, electronic reprints on demand)

(Staffan Björck, Stefan Franzén, Per Hjerpe, Karin Manhem, Annika Rosengren, Jörgen Thorn, Samuel Adamsson Eryd)

Presented as a poster on ESC Paris 2019. Part of a doctoral thesis 11/10 2019, http://hdl.handle.net/2077/60284

For peer review only

This work was supported by Närhälsan R&D Health Care, R&D Centre Gothenburg and Södra Bohuslän for postgraduate studies. This work was also supported by grants from the Swedish government under the Agreement concerning Research and Education of Doctors [grant number ALFGBG-717211], the Swedish Heart and Lung Foundation [grant number 2018-0366] and the Swedish Research Council [grant number 2018-02527].

Wordcount manuscript: 3,377

For peer review only

Abstract

Objectives

The protective effect of lipid lowering treatment for secondary prevention after coronary heart disease (CHD) has been well documented. Current guidelines recommend a target level for low density lipoprotein cholesterol (LDL-C) of ≤1.8 mmol/L. The aim was to describe lipid lowering treatment patterns and to provide an estimate of the potential reductions in cardiovascular disease (CVD) events with improved adherence to guidelines.

Design

Cross-sectional.

Setting

Primary care in a large Swedish region.

Participants

37,120 patients with CHD in a Swedish regional primary care quality register (QregPV), by 31 December 2015.

Primary and secondary outcome measures

For peer review only - http://bmjopen.bmj.com/site/about/guidelines.xhtml

Proportion of patients on statin treatment and proportion of patients achieving LDL-C ≤1.8 mmol/L. Estimated number of CVD events calculated for a) current treatment, b) improved treatment and c) lowered LDL-C, based upon applying rate reductions from meta-analyses of randomized trials to the potentially undertreated population. Risk estimation modelling was based on 52,042 patients in the same register on January 2011 followed for five years.

Results

Of 37,120 patients, 18% reached LDL-C ≤1.8 mmol/L and 32% were not on statin treatment. Based on individual risks, the estimated number of CVD events in the study group over 5 years was 9,209/37,120. If all patients without a statin or with less potent statin treatment were given atorvastatin 80 mg, an estimated reduction of CVD events by 14% (7,901 vs 9,209) was seen. If all patients achieved LDL-C ≤1.8 mmol/L the number of events was estimated to be reduced by 18% (7,577 vs 9,209).

For peer review only

Conclusion

One third of patients with CHD in primary care were not on lipid lowering treatment. Based on the assumption that included patients would react to statin therapy the same way as the patients in randomized trials, improved adherence to treatment guidelines could lead to a substantial reduction in new CVD events.

Wordcount abstract: 299

Keywords

cardiovascular disease, guideline adherence, primary health care, secondary prevention, statins, Sweden

Strengths and limitations of this study

The study was based on all patients with coronary heart disease in primary care in a Swedish region.

Data on comorbidities, filled prescriptions and outcomes were available for all patients.

Risk model calculations were based on the same regional population as the predictions.

Whether patients in our study react to statin therapy similarly as in the randomized trials in the meta-analysis used is not known.

There were missing data regarding BMI and smoking, known risk factors for cardiovascular disease.

Ethical approval

The regional Ethical Review Board at the University of Gothenburg

For peer review only

Introduction

Lowering of low density lipoprotein cholesterol (LDL-C) by statin treatment in patients with coronary heart disease (CHD) effectively reduces the risk of recurrent cardiovascular disease (CVD) events irrespective of age[1, 2]. Current evidence indicates an almost linear relationship between LDL-C level and risk of CVD[3, 4]. Although CVD mortality has decreased by over 60 % over the past few decades in Sweden, it still accounts for one out of every three deaths[5].

Low adherence to statin treatment is associated with a greater risk of death and new CVD events[6, 7]. The first years after a cardiovascular event, adherence to secondary preventive treatment is still high but diminishes over time [8, 9]. Adverse events are rare but nevertheless pose barriers for statin treatment both in patients and physicians[10-12].

Current European and Swedish guidelines recommend a treatment goal for LDL-C below 1.8 mmol/L in patients with established CHD[13, 14]. There has been a gradual improvement in the proportion of patients in Sweden reaching the LDL-C target one year after an acute myocardial infarction (AMI), and recent data suggests a figure of 65% in 2017[15]. Nevertheless, less is known about long-term adherence to current guidelines among patients with CHD in primary care or how an improvement in that regard affects morbidity.

The aim was to describe lipid lowering treatment patterns and to provide an estimate of the potential reductions in CVD events with improved adherence to guidelines.

Methods

We performed an observational cross-sectional register study of all primary care patients with CHD in a large Swedish region as of 31 December 2015.

For peer review only - http://bmjopen.bmj.com/site/about/guidelines.xhtml

Study basis

Västra Götaland (VGR) in south-west Sweden is a mixed urban and rural region with 1.7 million inhabitants (17% of the Swedish population). Since 2009, all individuals in the region have been enrolled at a primary care centre. The region has about 200 centres that are publicly funded, whether publicly or privately operated. Primary care centres report individual data for all patients with CHD to a regional primary care quality register (QregPV).

Databases

This study proceeded from information obtained by linking data from QregPV, a regional administrative health care database (Vega), the National Patient Register (NPR), the Prescribed Drug Register and the Cause of Death Register.

QregPV contains information about all primary care patients, irrespective of age or severity of disease, with a diagnosis of hypertension and/or CHD in VGR, including blood pressure, lipid levels, smoking, height and weight recorded during appointments and reported on a monthly basis. Starting in 2009, reporting to the register has been mandatory for all primary care units in VGR.

Vega covers all primary care in VGR since 2000. Data include diagnoses, dates and professional categories. NPR covers all hospital discharge diagnoses in Sweden since 1987, as well as outpatient appointments in specialised care since 2001. This study included NPR data since 1997, when the International Classification of Disease 10th revision (ICD-10) was adopted. The Prescribed Drug Register contains information about all prescriptions filled since 1 July 2005. The Cause of Death Register consists of information about underlying and contributing causes of death since 1961.

For peer review only - http://bmjopen.bmj.com/site/about/guidelines.xhtml

All patients in QregPV with a diagnosis of CHD entered from 1 January 2010 to 31 December 2015 were included. Diagnoses were defined according to ICD-10: CHD I20-I25, AMI I21, stroke I61, I63 - I64, type 2 diabetes mellitus E10-14, heart failure I50 and atrial fibrillation and flutter I48. CVD was defined as acute myocardial infarction (AMI) or stroke.

Patients

Proceeding from the patient file, we created two cohorts:

- Study cohort (2015) – All patients with CHD in QregPV on 31 December 2015 (index date). Previous comorbidity until 31 December 2015 and prescriptions filled up to 120 days before the index date were collected from NPR, Vega and the Prescribed Drug Register.
- Risk estimation cohort (2011) – 52,042 patients with CHD in QregPV on 1 January 2011 (index date). Previous comorbidity until the index date and new events corresponding to diagnoses of AMI and stroke until 31 December 2015 were added from NPR and Vega. Information about deaths were retrieved from the Cause of Death Register.

For both cohorts, medical data were collected in accordance with the last observation carried forward (LOCF) method. We used LOCF in order to include as many patients as possible since we considered using the last measured value a better approach then imputation or further exclusion of patients due to missing values. The latest entry of each variable was used up to 900 days before the index date.

For peer review only - http://bmjopen.bmj.com/site/about/guidelines.xhtml

For peer review only

Risk estimation

The risk estimation cohort was used to create a model for the risk of recurrent CVD and all-cause mortality based on individual data for age, sex, diabetes mellitus, history of heart failure and/or atrial fibrillation, stroke or AMI during the previous year and treatment with acetylic salicylic acid. Statin treatment was not included since it is one of the factors we assess. Smoking and BMI were not included due to a large number of missing values. Blood pressure was not included due to missing data in 2010 and the J-curved association described in observational studies[16].

Prediction

We calculated the individual risk of CVD and death from all causes, for patients in the study cohort, using the risk prediction model. We performed two separate adjusted predictions and evaluated the effect of:

- lowering LDL-C to 1.8 mmol/L for all patients with a higher level
- adding atorvastatin 40/80 mg to patients with no or less potent statin therapy and LDL-C > 1.8 mmol/L.

For risk reduction associated with lowering of LDL-C, data from Cholesterol Treatment Trialists' (CTT) collaboration were used, assuming that our patients react to statin therapy the same way as the patients included in those randomized trials . Overall risk reduction for any major vascular event was 22% (rate ratio 0.78; 95% CI 0.76-0.80) per mmol/L reduction in LDL-C based on almost 170,000 individuals[3]. Risk reduction values for separate outcomes in the CTT study differed. Risk reduction per mmol/L LDL-C reduction for all-cause mortality was 0.91 based on results from another CTT collaboration study[17]. Risk reduction with intensified statin treatment proceeded from expected percentage LDL-C lowering related to type of statin and dosage based on studies concerning statin effect on LDL-C level[18, 19].

For peer review only - http://bmjopen.bmj.com/site/about/guidelines.xhtml

Statistical methods

Descriptive statistics are presented as arithmetic mean and standard deviation for continuous variables and with frequencies and percentages for categorical variables. A Cox regression model was applied to the risk estimation cohort. The variables included in the model were age, sex, diabetes mellitus, history of heart failure, history of atrial fibrillation, treatment with acetyl salicylic acid and stroke or AMI during the previous year. Age was modelled by means of a smoothing spline-function with four degrees of freedom since the effect of age was not assumed to be linear. Sensitivity analyses with different models including BMI, smoking and systolic blood pressure were performed in order to test for robustness.

Time to event was right censored at death as end of follow up for total mortality and time to CVD. The time-at-risk for each patient was defined as the time before the first event over a period of five years.

The survival function and risk of having experienced an event over the past 5 years were calculated. Summing up individual risks yielded the total number of individuals predicted to have experienced an event over the past 5 years. Adjustments were made such that the cumulative hazard was recalculated based on an expected reduced risk for events related to lowering of LDL-C directly or expected lowering as a result of more intense statin treatment. The cumulative hazard of an event was reduced by 22% per mmol/L LDL-C lowering. This model did not take competing risks into account, assuming that all patients were alive after five years if they did not suffer an event.

The statistical analyses used R 3.4.0 and SAS version 9.4 (SAS Institute, Cary, NC).

For peer review only - http://bmjopen.bmj.com/site/about/guidelines.xhtml

Patient and public involvement

Patients or the public were not involved in the design, or conduct, or reporting, or dissemination plans of our research.

Results

Descriptive statistics for the study cohort are shown in Table 1. There was a total of 57,341 patients, 37,120 of whom had information about LDL-C at baseline. Fewer than 20% (6,747/37,120) achieved the LDL-C target of ≤ 1.8 mmol/L. Among the non-controlled patients, with LDL-C > 1.8 mmol/L, an average LDL-C reduction of 1.2 mmol/L (40%) would be required to reach the LDL-C target. The subset of patients with AMI or stroke in the previous year had a mean age of 74.2 (10.5); 78% received statin treatment and 25% reached the LDL-C target.

Table 1. Characteristics of patients in the study cohort.

Variable	All patients (n = 37,120)	LDL-C <= 1.8 mmol/L (n= 6,747)	LDL-C > 1.8 mmol/L (n= 30,373)
Age	73.0 (10.1)	73.2 (9.9)	73.0 (10.1)
Sex (female)	13,585 (36.6%)	1,897 (28.1%)	11,688 (38.5%)
Smoking*	4,221 (12.7%)	755 (12.3%)	3,466 (12.7%)
SBP (mmHg)	132.8 (16.1)	130.6 (15.6)	133.3 (16.2)
DBP (mmHg)	75.3 (10.7)	73.9 (10.7)	75.6 (10.7)
Total cholesterol (mmol/L)*	4.5 (1.2)	3.4 (0.7)	4.8 (1.1)
LDL-C (mmol/L)	2.7 (1.0)	1.5 (0.3)	3.0 (0.9)
Triglyceride (mmol/L)*	1.6 (0.9)	1.5 (1.0)	1.6 (0.9)

For peer review only - http://bmjopen.bmj.com/site/about/guidelines.xhtml

Variable	All patients (n = 37,120)	LDL-C <= 1.8 mmol/L (n= 6,747)	LDL-C > 1.8 mmol/L (n= 30,373)
Hypertension	30,869 (83.2%)	5,649 (83.7%)	25,220 (83.0%)
Diabetes	12,544 (33.8%)	3,116 (46.2%)	9,428 (31.0%)
CHD	37,120 (100.0%)	6,747 (100.0%)	30,373 (100.0%)
AMI	16,742 (45.1%)	3,740 (55.4%)	13,002 (42.8%)
AMI past year	1,544 (4.2%)	441 (6.5%)	1,103 (3.6%)
Stroke	4,452 (12.0%)	921 (13.7%)	3,531 (11.6%)
Stroke past year	1,292 (3.5%)	266 (3.9%)	1,026 (3.4%)
CVD	19,183 (51.7%)	4,177 (61.9%)	15,006 (49.4%)
Heart failure	8,888 (23.9%)	1,951 (28.9%)	6,937 (22.8%)
Atrial fibrillation	8,257 (22.2%)	1,807 (26.8%)	6,450 (21.2%)
Dementia	1,363 (3.7%)	224 (3.3%)	1,139 (3.8%)
ASA	23,431 (63.1%)	4,426 (65.6%)	19,005 (62.6%)
Statin	25,160 (67.8%)	5,735 (85.0%)	19,425 (64.0%)
Simvastatin	12,820 (34.5%)	2,810 (41.6%)	10,010 (33.0%)
Pravastatin	286 (0.8%)	20 (0.3%)	266 (0.9%)
Atorvastatin	11,424 (30.8%)	2,723 (40.4%)	8,701 (28.6%)
Rosuvastatin	998 (2.7%)	235 (3.5%)	763 (2.5%)
Other lipid lowering drugs	1,231 (3.3%)	227 (3.4%)	1,004 (3.3%)
Ezetimib	953 (2.6%)	175 (2.6%)	778 (2.6%)

*Mean (SD) and frequencies (%). Abbreviations: SBP: Systolic blood pressure; DBP: Diastolic blood pressure; LDL-C: Low density lipoprotein cholesterol; CHD: coronary heart disease; AMI: acute myocardial infarction; CVD: cardiovascular disease; ASA: acetylic salicylic acid. *Missing data: Smoking 3,753, SBP 616, DBP 625, Total cholesterol 1,510, Triglyceride 5,366. For other variables there is no missing data.*

For peer review only - http://bmjopen.bmj.com/site/about/guidelines.xhtml

Statin treatment was most prevalent for age 50-75, approximately 70% of whom filled a prescription, see Figure 1. Prescriptions filled for statins diminish rapidly after the age of 80.

Figure 1.

LDL-C for uncontrolled patients was predicted to decrease from a mean of 3.0 to 2.2 mmol/L if all patients received atorvastatin 40 mg and to 2.0 mmol/L with atorvastatin 80 mg. For all patients in the study cohort the corresponding levels were 2.7 to 2.1 and 1.9 mmol/L, see LDL-C and predicted LDL-C distribution in Figure 2.

Figure 2.

Total predicted CVD events for 5 years in the study cohort was 9,209 (24.8%), see Figure 3. A decrease of LDL-C to the target (≤ 1.8 mmol/l) or intensified statin treatment (all patients with less efficient therapy receive atorvastatin 80 mg) was estimated to result in a reduction of expected CVD events for 5 years by 1,632 and 1,308. This corresponds to a decreased event risk of 18% and 14%. Including only the patients with LDL-C > 1.8, the corresponding estimated reductions were 22% and 18%. All-cause mortality for 5 years was estimated to decline by 6.4 % (534/8,344) when LDL-C was lowered to ≤ 1.8 mmol/L.

Figure 3.

For peer review only - http://bmjopen.bmj.com/site/about/guidelines.xhtml

Sensitivity analyses by adding variables showed that the model was robust (supplementary table I). When BMI and smoking status were included, the number of patients in the risk estimation cohort was reduced, from 52,042 to 9,254. The proportion of patients who were predicted to suffer a CVD event was decreased from 25% to 22% but the relative reduction in number of events was the same. The results were similar without including BMI and smoking status in the model. The same was true if the risk estimation model was based on the 16,577 patients with LDL-C (although LDL-C was not in the model). When systolic blood pressure was included in the model, the risk estimation cohort was decreased to 35,989 and the outcomes were relatively unchanged. Inclusion of statin treatment (0/1) in the model did not lead to any changes in the results.

The proportion of patients predicted to experience a CVD event was 24.6 % (2,937/11,960) among non-statin users and 24.9 % (6,271/25,160) among statin users, see Figure 4. If all patients were prescribed 80 mg atorvastatin, the predicted proportion to experience a CVD event was reduced by 32.9 % (967) among non-statin users and 5.4 % (339) among statin users. The patients with statin treatment were younger (72.3 vs 74.5) and consisted of more men (68% vs 54%), had a diabetes diagnosis to a larger extent (36% vs 29%) and more often had a stroke or AMI during the past year (4-5% vs 2.5%) (supplementary table II).

Figure 4.

Other lipid-lowering drugs were rarely used, 953/37,120 (2.6%) only 442 of whom were not also on statin. Patients receiving other lipid-lowering drugs had slightly higher LDL-C than the rest of the study population.

For peer review only

For peer review only - http://bmjopen.bmj.com/site/about/guidelines.xhtml

Discussion

Summary

One third of our study population had no statin treatment and fewer than 1/5 reached the recommended LDL-C target of 1.8 mmol/L. Using data on the effect of statins from randomized trials, the number of CVD events was estimated to be substantially reduced if patients with no/low-dose statin were given atorvastatin 80 mg and even more so if all patients reached the LDL-C target. The largest gain seemed to result from initiating treatment in patients who are not receiving statin.

Strengths and limitations

The main strength of our study was the large number of primary care patients with CHD. Previous studies were often based on a post-MI population from secondary care registers. Another strength is the completeness of data concerning comorbidities and filled prescriptions. We based our risk model calculations on the same regional population as the predictions.

Information about LDL-C level was missing for many patients in the study cohort. These patients were older, diagnosed more often with atrial fibrillation and heart failure, less with diabetes and not as likely to be receiving statin treatment. Inclusion of them would probably have increased the predicted number of saved events. BMI, smoking and blood pressure were not included in the model due to the quantity of missing values in the risk estimation cohort. Sensitivity analyses showed that the results remained approximately the same without these variables.

It has been shown that compliance per se (placebo or active treatment) is associated with outcomes[20]. We assumed that all patients benefit proportionally from better adherence and improved treatment. A limitation of our study is therefore that included patients with suboptimal statin treatment might not respond to statin therapy in the same way as

For peer review only - http://bmjopen.bmj.com/site/about/guidelines.xhtml

participants in the randomized trials in the meta-analysis used for risk reduction calculations. Furthermore, patients with no statin treatment could either have no prescription by a physician or be non-compliant. We were not able to adjust for this difference but since our aim was not to estimate causal effects but to get an estimation of the effects of a changed treatment pattern, we consider our results still valid.

We did not take competing risks into account and assumed that no patient had died from other causes during the follow-up period. Patients in our risk estimation cohort who died before a potential event were right censored and 9.2% died from other causes without a previous event. Our model appears to slightly overestimate the number of CVD events, but the percentage decrease is not necessarily affected.

For peer review only

Comparison with existing literature

A recent Swedish study described short-term adherence to statins and potential effects of improved treatment among 5,904 post-MI patients[21]. The study found an estimated 805-2,262 CVD events in over 10 years among those who did not reach LDL-C target and predicted that a reduction of LDL-C to 1.8 mmol/L would lower the number by 132-343 (15-16%). The patients in that study were younger, which might explain the smaller predicted reduction in events.

LDL-C is monitored one year after AMI in the Swedish national quality register (SEPHIA). In 2015, mean LDL-C was 1.99 mmol/L and 51% had LDL-C < 1.8 mmol/L[22]. Lipid lowering treatment after AMI is gradually increasing in Sweden[15]. A Norwegian study of 42,707 patients discharged from hospital after AMI in 2009-2013 reported that 84% were being treated with statins one year later[8]. Mean LDL-C was 2.7 mmol/L in our study cohort and only 18% had LDL-C ≤ 1.8 mmol/L also including those younger than 75 as those in SEPHIA. This difference between improved treatment in the short term and our finding of poor control in the long term may be due to poorer

For peer review only - http://bmjopen.bmj.com/site/about/guidelines.xhtml

adherence to lipid-lowering treatment over time and the fact that new guidelines are applied to patients with a recent AMI event. Poor long-term adherence has been demonstrated earlier with discontinuation rates of 67% over 5 years[23]. The probability of persistence has increased over the years but remains low already a few years after initiation of treatment[24]. Since our study is based on an unselected regional primary care patient population, it seems plausible that the results are generalisable to regions and countries with a similar population.

Over the past few years, statin treatment has proven to be safe and effective among the aged as well, but the proportion of patients in our study receiving it diminishes after the age of 75[2]. The importance of continued statin treatment is further stressed by the fact that LDL-C remains an important risk factor for CVD even at high age as opposed to other traditional risk factors [1].

Our cohort consists of primary care patients with a CHD diagnosis. It has been shown that patients in primary care with known CVD from diagnoses in hospital but without a registered one in primary care receive less statin treatment[25]. In light of these results, our study presumably underestimates the potential event reduction by means of improved treatment.

The reasons for non-adherence to guidelines may depend on the physician, patient or organisational factors. A recent Swedish study showed that patients with concomitant use of other cardio-protective medications or smoking had higher adherence to refill and lipid-lowering treatment[26]. Two Danish studies found adherence to statin treatment to be lower among patients with a more risk-seeking attitude and physicians who did not assess treatment after initiation[27, 28]. The reasons that physicians do not adhere to lipid-lowering guidelines may be the same as for the blood pressure target: acceptance of higher values than recommended, competing medical problems, etc.[29]. Regardless of reasons

For peer review only

For peer review only - http://bmjopen.bmj.com/site/about/guidelines.xhtml

for non-adherence, our study shows that there is room for improvement of secondary preventive lipid lowering therapy.

Implications for research and/or practice

In this unselected primary care population of patients with CHD in a large Swedish region, a high proportion of patients do not reach LDL-C target. Assuming the same benefits of intensified statin treatment as in the CTT meta-analyses, there is a significant potential to reduce the number of events if treatment guidelines were to be followed to a greater extent. The results of this cross-sectional study support further studies of the effect of increased compliance with recommendations.

For peer review only

Funding

The authors disclosed receipt of the following financial support for the research, authorship, and/or publication of this article: This work was supported by Närhälsan R&D Health Care, R&D Centre Gothenburg and Södra Bohuslän for postgraduate studies. This work was also supported by grants from the Swedish government under the Agreement concerning Research and Education of Doctors [grant number ALFGBG-717211], the Swedish Heart and Lung Foundation [grant number 2018-0366] and the Swedish Research Council [grant number 2018-02527].

Declaration of Conflicting Interests

SAE is employed by AstraZeneca since September 2019 but was affiliated to University of Gothenburg during the main part of the study.

Author contributions

HÖ, SB, SF, PH, KM, AR, JT and SAE contributed to the conception and design of the work. All authors contributed to the acquisition, analysis, or interpretation of data for the work. HÖ drafted the manuscript. All authors critically revised the manuscript. All authors gave final approval and agree to be accountable for all aspects of work ensuring integrity and accuracy.

For peer review only - http://bmjopen.bmj.com/site/about/guidelines.xhtml

Data sharing statement

No additional unpublished data are available.

For peer review only - http://bmjopen.bmj.com/site/about/guidelines.xhtml

References

1. Lind L, Sundstrom J, Arnlov J, Lampa E. Impact of Aging on the Strength of Cardiovascular Risk Factors: A Longitudinal Study Over 40 Years. J Am Heart Assoc. 2018;7(1).

2. Cholesterol Treatment Trialists C, Armitage J, Baigent C, Barnes E, Betteridge D. Efficacy and safety of statin therapy in older people: a meta-analysis of individual participant data from 28 randomised controlled trials. Lancet. 2019;393(10170):407-15.

3. Cholesterol Treatment Trialists C, Baigent C, Blackwell L, Emberson J, Holland LE, Reith C, Bhala N, Peto R, Barnes EH, Keech A, Simes J, Collins R. Efficacy and safety of more intensive lowering of LDL cholesterol: a meta-analysis of data from 170,000 participants in 26 randomised trials. Lancet. 2010;376(9753):1670-81.

4. Ference BA, Ginsberg HN, Graham I, Ray KK, Packard CJ, Bruckert E, Hegele RA, Krauss RM, Raal FJ, Schunkert H, Watts GF, Boren J, Fazio S, Horton JD, Masana L, Nicholls SJ, Nordestgaard BG, van de Sluis B, Taskinen MR, Tokgozoglu L, Landmesser U, Laufs U, Wiklund O, Stock JK, Chapman MJ, Catapano AL. Low-density lipoproteins cause atherosclerotic cardiovascular disease. 1. Evidence from genetic, epidemiologic, and clinical studies. A consensus statement from the European Atherosclerosis Society Consensus Panel. Eur Heart J. 2017;38(32):2459-72.

5. Statistik om dödsorsaker 2017 [Cause of Death statistics 2017]. https://www.socialstyrelsen.se/Lists/Artikelkatalog/Attachments/21101/2018-10-17.pdf: Social Board of Health and Welfare, 2018.

6. Rodriguez F, Maron DJ, Knowles JW, Virani SS, Lin S, Heidenreich PA. Association of Statin Adherence With Mortality in Patients With Atherosclerotic Cardiovascular Disease. JAMA Cardiol. 2019;4(3):206-13.

7. Banach M, Stulc T, Dent R, Toth PP. Statin non-adherence and residual cardiovascular risk: There is need for substantial improvement. Int J Cardiol. 2016;225:184-96.

For peer review only

8. Halvorsen S, Jortveit J, Hasvold P, Thuresson M, Oie E. Initiation of and long-term adherence to secondary preventive drugs after acute myocardial infarction. BMC Cardiovasc Disord. 2016;16:115.

9. Ofori-Asenso R, Jakhu A, Zomer E, Curtis AJ, Korhonen MJ, Nelson M, Gambhir M, Tonkin A, Liew D, Zoungas S. Adherence and Persistence Among Statin Users Aged 65 Years and Over: A Systematic Review and Meta-analysis. J Gerontol A Biol Sci Med Sci. 2018;73(6):813-9.

10. Tramacere I, Boncoraglio GB, Banzi R, Del Giovane C, Kwag KH, Squizzato A, Moja L. Comparison of statins for secondary prevention in patients with ischemic stroke or transient ischemic attack: a systematic review and network meta-analysis. BMC Med. 2019;17(1):67.

11. Tobert JA, Newman CB. Statin tolerability: In defence of placebo-controlled trials. Eur J Prev Cardiol. 2016;23(8):891-6.

12. Kruger K, Leppkes N, Gehrke-Beck S, Herrmann W, Algharably EA, Kreutz R, Heintze C, Filler I. Improving long-term adherence to statin therapy: a qualitative study of GPs' experiences in primary care. Br J Gen Pract. 2018;68(671):e401-e7.

13. To prevent atherosclerotic cardiovascular disease with drugs - treatment recommendations. Stockholm. Sweden. Medical Product Agency; updated 2014; Available at: https://lakemedelsverket.se/upload/halso-och-sjukvard/behandlingsrekommendationer/Att_forebygga_aterosklerotisk_hjart-karlsjukdom_med%20_lakemedel_behandlingsrekommendation.pdf (accessed 7/12 2018).

14. Authors/Task Force M, Piepoli MF, Hoes AW, Agewall S, Albus C, Brotons C, Catapano AL, Cooney MT, Corra U, Cosyns B, Deaton C, Graham I, Hall MS, Hobbs FD, Lochen ML, Lollgen H, Marques-Vidal P, Perk J, Prescott E, Redon J, Richter DJ, Sattar N, Smulders Y, Tiberi M, van der Worp HB, van Dis I, Verschuren WM, Additional Contributor: Simone B, Document R, De Backer G, Roffi M, Aboyans V, Bachl N, Bueno H, Carerj S, Cho L, Cox J, De Sutter J, Egidi G, Fisher M, Fitzsimons D, Franco OH, Guenoun M, Jennings C, Jug B, Kirchhof P, Kotseva K, Lip GY, Mach F, Mancia G, Bermudo FM, Mezzani A, Niessner A, Ponikowski P, Rauch B, Ryden L, Stauder A, Turc G, Wiklund O, Windecker

For peer review only

S, Zamorano JL. 2016 European Guidelines on cardiovascular disease prevention in clinical practice: The Sixth Joint Task Force of the European Society of Cardiology and Other Societies on Cardiovascular Disease Prevention in Clinical Practice (constituted by representatives of 10 societies and by invited experts): Developed with the special contribution of the European Association for Cardiovascular Prevention & Rehabilitation (EACPR). Eur J Prev Cardiol. 2016;23(11):NP1-NP96. Epub 2016/06/30.

15. Swedeheart. SWEDEHEART Annual Report 2017. Stockholm: 2017.

16. Adamsson Eryd S, Gudbjornsdottir S, Manhem K, Rosengren A, Svensson AM, Miftaraj M, Franzen S, Bjorck S. Blood pressure and complications in individuals with type 2 diabetes and no previous cardiovascular disease: national population based cohort study. BMJ. 2016;354:i4070. Epub 2016/08/06.

17. Cholesterol Treatment Trialists C, Fulcher J, O'Connell R, Voysey M, Emberson J, Blackwell L, Mihaylova B, Simes J, Collins R, Kirby A, Colhoun H, Braunwald E, La Rosa J, Pedersen TR, Tonkin A, Davis B, Sleight P, Franzosi MG, Baigent C, Keech A. Efficacy and safety of LDL-lowering therapy among men and women: meta-analysis of individual data from 174,000 participants in 27 randomised trials. Lancet. 2015;385(9976):1397-405.

18. Law MR, Wald NJ, Rudnicka AR. Quantifying effect of statins on low density lipoprotein cholesterol, ischaemic heart disease, and stroke: systematic review and meta-analysis. BMJ. 2003;326(7404):1423.

19. NICE. NICE Guidance - Cardiovascular disease: risk assessment and reduction, including lipid modification. London: The National Institute for Health and Care Excellence (NICE); 2014 updated 2016; Available at: https://www.nice.org.uk/guidance/cg181/chapter/appendix-a-grouping-of-statins (accessed 14/11 2018).

20. Coronary Drug Project Research G. Influence of adherence to treatment and response of cholesterol on mortality in the coronary drug project. N Engl J Med. 1980;303(18):1038-41.

For peer review only

For peer review only - http://bmjopen.bmj.com/site/about/guidelines.xhtml

21. Journath G, Hambraeus K, Hagstrom E, Pettersson B, Lothgren M. Predicted impact of lipid lowering therapy on cardiovascular and economic outcomes of Swedish atherosclerotic cardiovascular disease guideline. BMC Cardiovasc Disord. 2017;17(1):224.

22. SWEDEHEART. SWEDEHEART Annual report 2015. Stockholm: 2015.

23. Ofori-Asenso R, Ilomaki J, Tacey M, Zomer E, Curtis AJ, Bell JS, Zoungas S, Liew D. Patterns of statin use and long-term adherence and persistence among older adults with diabetes. J Diabetes. 2018;10(9):699-707.

24. Chen ST, Huang ST, Shau WY, Lai CL, Li JZ, Fung S, Tse VC, Lai MS. Long-term statin adherence in patients after hospital discharge for new onset of atherosclerotic cardiovascular disease: a population-based study of real world prescriptions in Taiwan. BMC Cardiovasc Disord. 2019;19(1):62.

25. Dahlgren C, Geary L, Hasselstrom J, Rehnberg C, Schenck-Gustafsson K, Wandell P, Euler MV. Recording a diagnosis of stroke, transient ischaemic attack or myocardial infarction in primary healthcare and the association with dispensation of secondary preventive medication: a registry-based prospective cohort study. BMJ Open. 2017;7(9):e015723.

26. Karlsson SA, Hero C, Eliasson B, Franzen S, Svensson AM, Miftaraj M, Gudbjornsdottir S, Eeg-Olofsson K, Andersson Sundell K. Refill adherence and persistence to lipid-lowering medicines in patients with type 2 diabetes: A nation-wide register-based study. Pharmacoepidemiol Drug Saf. 2017;26(10):1220-32.

27. Barfoed BL, Paulsen MS, Christensen PM, Halvorsen PA, Jarbol DE, Larsen ML, Munch MR, Sondergaard J, Nielsen JB. Associations between patients' adherence and GPs' attitudes towards risk, statin therapy and management of non-adherence--a survey and register-based study. Fam Pract. 2016;33(2):140-7.

28. Barfoed BL, Paulsen MS, Christensen PM, Halvorsen PA, Kjaer T, Larsen ML, Larsen PV, Nielsen JB, Sondergaard J, Jarbol DE. Associations between patients' risk attitude and their

For peer review only

For peer review only - http://bmjopen.bmj.com/site/about/guidelines.xhtml

adherence to statin treatment - a population based questionnaire and register study. BMC Fam Pract. 2016;17:28.

29. Oliveria SA, Lapuerta P, McCarthy BD, L'Italien GJ, Berlowitz DR, Asch SM. Physician-related barriers to the effective management of uncontrolled hypertension. Arch Intern Med. 2002;162(4):413-20.

For peer review only

For peer review only - http://bmjopen.bmj.com/site/about/guidelines.xhtml

Legends

Figure 1. Statin treatment by age. Included ages with more than 100 observations. The number of patients below the age of 50 are very few (603/37,120) as are the patients over the age of 90 (925/37,120).

Figure 2. LDL-C distribution in all patients in the study cohort with current treatment and adjusted treatment defined as if all patients received atorvastatin 80 mg (if less intense treatment before and LDL-C > 1.8 mmol/L).

Figure 3. Estimated number of events in the study cohort (37,120 patients) with registered levels of LDL-C and current treatment as well as when modelling a reduction in LDL-C to 1.8 mmol/L for all patients with higher LDL-C or an intensified statin lowering treatment with 80 mg atorvastatin for those with a less efficient treatment.

Figure 4. Estimated number of events in the study cohort with registered levels of LDL-C and current treatment separated in patients with and without current statin treatment and the predicted number of events when applying a more intense statin treatment to all patients with a less efficient treatment.

For peer review only - http://bmjopen.bmj.com/site/about/guidelines.xhtml

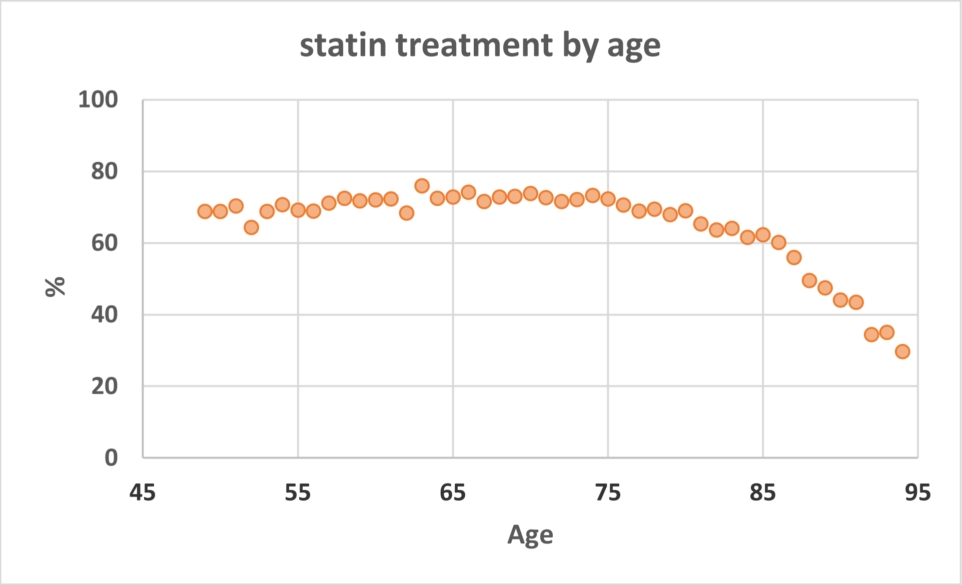

1 (1)

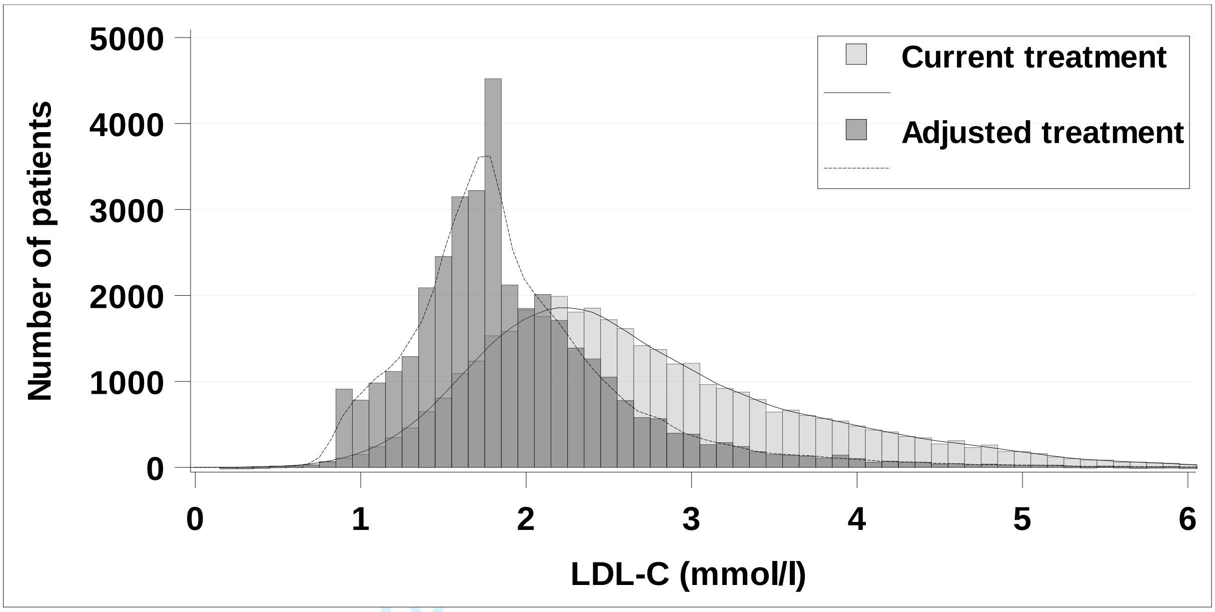

For peer review only - http://bmjopen.bmj.com/site/about/guidelines.xhtml

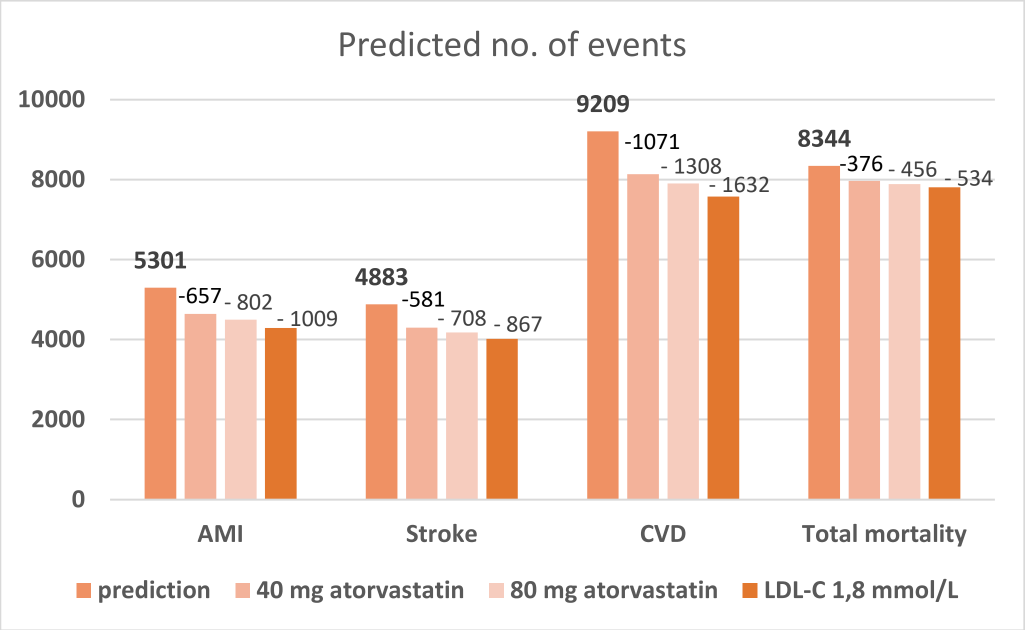

review only

1 (**1**)

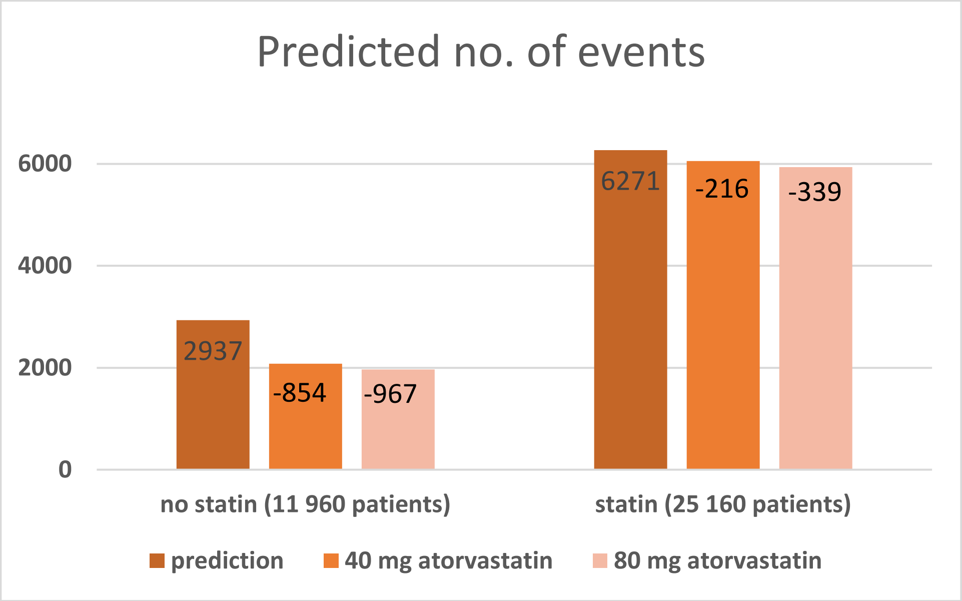

For peer review only

For peer review only - http://bmjopen.bmj.com/site/about/guidelines.xhtml

Supplementary information – Adherence to lipid-lowering guidelines for secondary prevention and potential reduction in CVD events in Swedish primary care – a cross-sectional study. Ödesjö et al

Table I. Sensitivity analyses

	Percentage estimated events	Percentage estimated events with LDL-C < 1.8 mmol/L	Reduction in number of events
**Model presented in the manuscript**	24.8%	20.4%	17.7%
**Data with BMI/smoking[1]**	22.1%	18.3%	17.1%
**Data with LDL-C[2]**	22.2%	18.3%	17.6%
**With SBP in model[3]**	23.9%	19.7%	17.7%
**With statin in model[4]**	24.6%	20.2%	18.0%

[1] Calculations based on the patients in the risk and study cohorts with information on BMI and smoking. The same risk model was applied as in the presented material as well as a model including BMI and smoking. Both models resulted in similar percentages.

[2] Calculations based on the patients in the risk and study cohorts with information on LDL-C. The same risk model applied as in the presented material.

[3] Calculations based on the same risk and study cohorts but with SBP added to the model, patients without information on SBP were thereby not included.

[4] Calculations based on the same risk and study cohorts but with statin added to the model.

For peer review only

Table II. Characteristics of patients in the study cohort divided by statin treatment or not.

Variable	No statin treatment (n= 11960)	Statin treatment (n= 25160)
Age	74.5 (11.0)	72.3 (9.5)
Sex (female)	5,529 (46.2%)	8,056 (32.0%)
Smoking	1,300 (12.5%)	2,921 (12.7%)
SBP (mmHg)	134.3 (17.0)	132.1 (15.6)
DBP (mmHg)	76.1 (11.2)	74.9 (10.4)
Total cholesterol (mmol/L)	5.2 (1.3)	4.2 (1.0)
LDL-C (mmol/L)	3.3 (1.1)	2.4 (0.8)
Triglyceride (mmol/L)	1.6 (0.9)	1.5 (0.9)
Hypertension	9,858 (82.4%)	21,011 (83.5%)
Diabetes	3488 (29.2%)	9056 (36.0%)
CHD	11,960 (100.0%)	25,160 (100.0%)
AMI	4,001 (33.5%)	12,741 (50.6%)
AMI past year	285 (2.4%)	1,259 (5.0%)
Stroke	1,315 (11.0%)	3,137 (12.5%)
Stroke past year	323 (2.7%)	969 (3.9%)
CVD	4,798 (40.1%)	14,385 (57.2%)

For peer review only

Heart failure	3,070 (25.7%)	5,818 (23.1%)
Atrial fibrillation	2,853 (23.9%)	5,404 (21.5%)
Dementia	589 (4.9%)	774 (3.1%)
ASA	5402 (45.2%)	18029 (71.7%)
Statin		25160 (100.0%)
Simvastatin		12820 (51.0%)
Pravastatin		286 (1.1%)
Atorvastatin		11424 (45.4%)
Rosuvastatin		998 (4.0%)
Other lipid lowering drugs	442 (3.7%)	789 (3.1%)
Ezetimib	277 (2.3%)	676 (2.7%)

Mean (SD) and frequencies (%). Abbreviations: SBP: Systolic blood pressure; DBP: Diastolic blood pressure; LDL-C: Low density lipoprotein cholesterol; CHD: coronary heart disease; AMI: acute myocardial infarction; CVD: cardiovascular disease; ASA: acetylic salicylic acid.

For peer review only

STROBE 2007 (v4) Statement—Checklist of items that should be included in reports of *cross-sectional studies*

Section/Topic	Item #	Recommendation	Reported on page #
**Title and abstract**	1	(*a*) Indicate the study's design with a commonly used term in the title or the abstract	1
		(*b*) Provide in the abstract an informative and balanced summary of what was done and what was found	3-4
**Introduction**			
Background/rationale	2	Explain the scientific background and rationale for the investigation being reported	6
Objectives	3	State specific objectives, including any prespecified hypotheses	6
**Methods**			
Study design	4	Present key elements of study design early in the paper	8
Setting	5	Describe the setting, locations, and relevant dates, including periods of recruitment, exposure, follow-up, and data collection	7
Participants	6	(*a*) Give the eligibility criteria, and the sources and methods of selection of participants	8
Variables	7	Clearly define all outcomes, exposures, predictors, potential confounders, and effect modifiers. Give diagnostic criteria, if applicable	9
Data sources/ measurement	8*	For each variable of interest, give sources of data and details of methods of assessment (measurement). Describe comparability of assessment methods if there is more than one group	7-8
Bias	9	Describe any efforts to address potential sources of bias	*Yes*
Study size	10	Explain how the study size was arrived at	
Quantitative variables	11	Explain how quantitative variables were handled in the analyses. If applicable, describe which groupings were chosen and why	9-10
Statistical methods	12	(*a*) Describe all statistical methods, including those used to control for confounding	9-10
		(*b*) Describe any methods used to examine subgroups and interactions	NA
		(*c*) Explain how missing data were addressed	9
		(*d*) If applicable, describe analytical methods taking account of sampling strategy	NA
		(*e*) Describe any sensitivity analyses	10
**Results**			

For peer review only

Participants	13*	(a) Report numbers of individuals at each stage of study—eg numbers potentially eligible, examined for eligibility, confirmed eligible, included in the study, completing follow-up, and analysed	10
		(b) Give reasons for non-participation at each stage	10
		(c) Consider use of a flow diagram	
Descriptive data	14*	(a) Give characteristics of study participants (eg demographic, clinical, social) and information on exposures and potential confounders	11
		(b) Indicate number of participants with missing data for each variable of interest	12
Outcome data	15*	Report numbers of outcome events or summary measures	13
Main results	16	(*a*) Give unadjusted estimates and, if applicable, confounder-adjusted estimates and their precision (eg, 95% confidence interval). Make clear which confounders were adjusted for and why they were included	NA
		(*b*) Report category boundaries when continuous variables were categorized	NA
		(*c*) If relevant, consider translating estimates of relative risk into absolute risk for a meaningful time period	
Other analyses	17	Report other analyses done—eg analyses of subgroups and interactions, and sensitivity analyses	13
**Discussion**			
Key results	18	Summarise key results with reference to study objectives	14
Limitations	19	Discuss limitations of the study, taking into account sources of potential bias or imprecision. Discuss both direction and magnitude of any potential bias	14-15
Interpretation	20	Give a cautious overall interpretation of results considering objectives, limitations, multiplicity of analyses, results from similar studies, and other relevant evidence	15-17
Generalisability	21	Discuss the generalisability (external validity) of the study results	17
**Other information**			
Funding	22	Give the source of funding and the role of the funders for the present study and, if applicable, for the original study on which the present article is based	17

*Give information separately for cases and controls in case-control studies and, if applicable, for exposed and unexposed groups in cohort and cross-sectional studies.

Note: An Explanation and Elaboration article discusses each checklist item and gives methodological background and published examples of transparent reporting. The STROBE checklist is best used in conjunction with this article (freely available on the Web sites of PLoS Medicine at http://www.plosmedicine.org/, Annals of Internal Medicine at http://www.annals.org/, and Epidemiology at http://www.epidem.com/). Information on the STROBE Initiative is available at www.strobe-statement.org.

For peer review only
